# Supplementary material for: Evaluation of efficacy and safety of AAV8-ΔC4ATP7B gene therapy in a mutant mouse model of Wilson’s disease
Source: Mol Ther Methods Clin Dev. 2025 Feb 13;33(1):101435. doi: 10.1016/j.omtm.2025.101435 (PMC11919453; doi:10.1016/j.omtm.2025.101435)
Supplement: Document S1. Figures S1–S6 and Tables S1 and S2 [file mmc1.pdf]

OMTM, Volume *33*

Supplemental information

Evaluation of efficacy and safety of AAV8-ΔC4ATP7B gene therapy in a mutant mouse model of Wilson's disease

Chunhua Zeng, Yunting Lin, Xinshuo Lu, Shehong Chen, Yan Xia, Kangdi Zhang, Yongxian Shao, Zhihong Guan, Rong Du, Zongcai Liu, Mingqi Zhao, Xiaoling Jiang, Yanna Cai, Taolin Li, Xueying Su, Yaoyong Chen, Xiaoyan Dong, Wen Zhang, Li Liu, and Wenhao Zhou

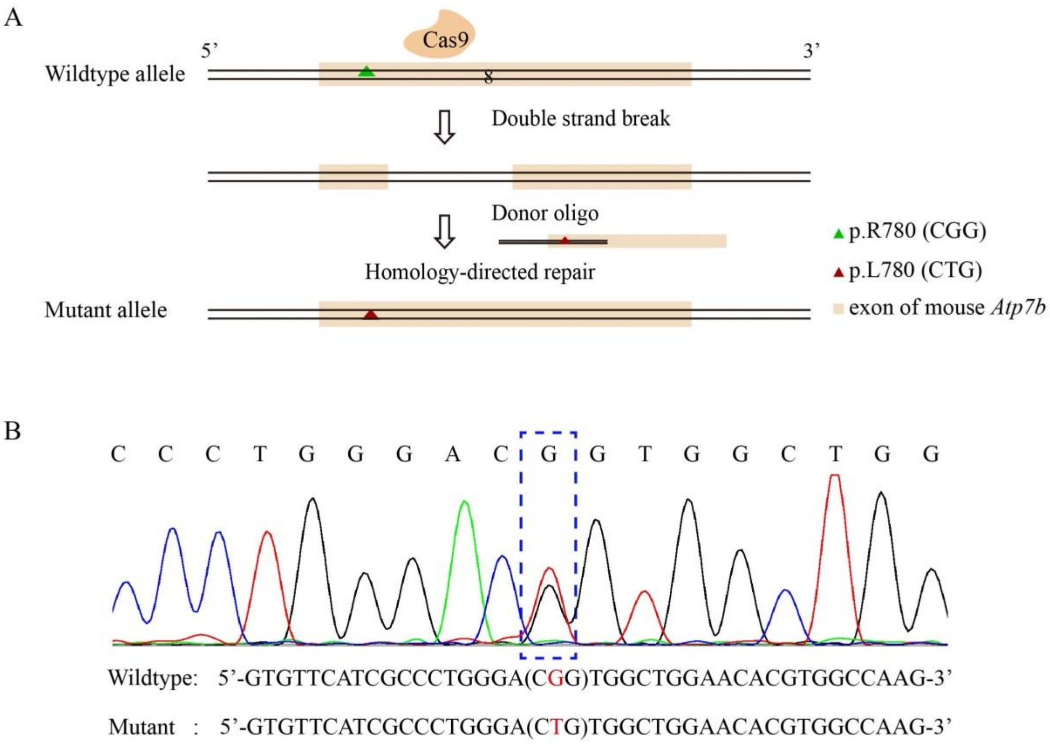

Figure S1. Generation of a C57BL/6 mouse model with *Atp7b*$^{\mathbf{R780L}}$ variant.

(A) Schematic diagram on the generation of the *Atp7b*$^{\mathrm{KI/KI}}$ mouse model. (B) Confirmation of the *Atp7b*$^{\mathrm{R780L}}$ variant by Sanger sequencing.

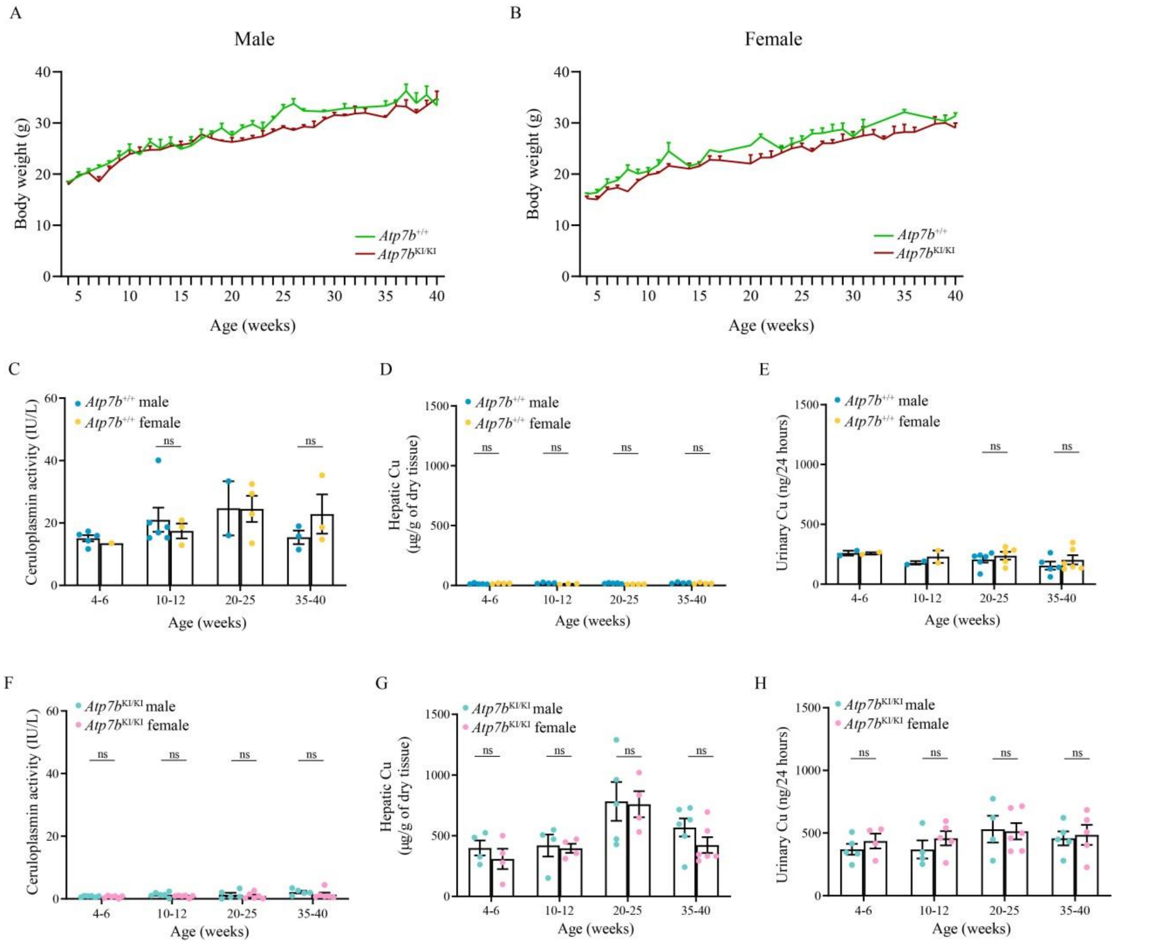

Figure S2. The weight gain and Cu metabolism biomarkers of *Atp7b*$^{KI/KI}$ and *Atp7b*$^{+/+}$ mice.

(A) Body weight in male *Atp7b*$^{KI/KI}$ and *Atp7b*$^{+/+}$ mice. (B) Body weight in female *Atp7b*$^{KI/KI}$ and *Atp7b*$^{+/+}$ mice. (C) Serum ceruloplasmin levels in *Atp7b*$^{+/+}$ mice. (D) Hepatic Cu content in *Atp7b*$^{+/+}$ mice. (E) Urinary Cu levels in *Atp7b*$^{+/+}$ mice. (F) Serum ceruloplasmin levels in *Atp7b*$^{KI/KI}$ mice. (G) Hepatic Cu content in *Atp7b*$^{KI/KI}$ mice. (H) Urinary Cu levels in *Atp7b*$^{KI/KI}$ mice.

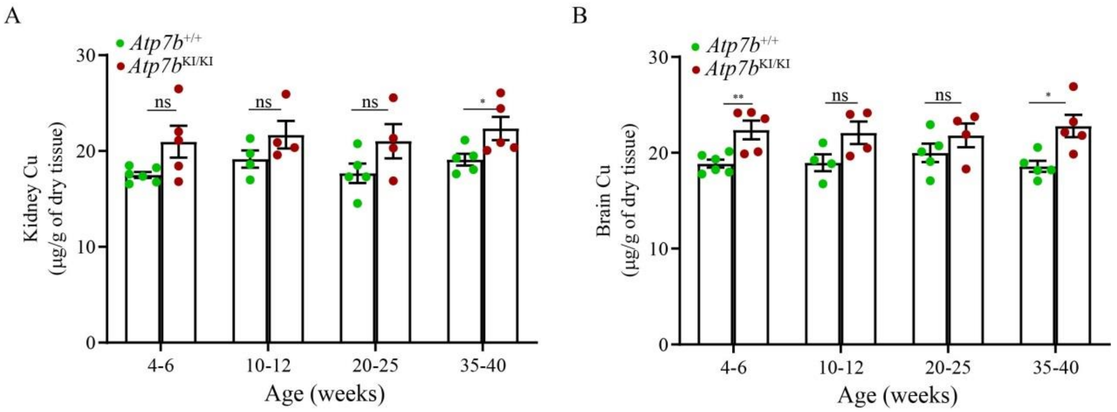

Figure S3. Kidney and brain Cu content in *Atp7b*$^{KI/KI}$ mice.

(A) Comparison of kidney Cu content between *Atp7b*$^{KI/KI}$ and *Atp7b*$^{+/+}$ mice. (B) Comparison of brain Cu content between *Atp7b*$^{KI/KI}$ and *Atp7b*$^{+/+}$ mice.

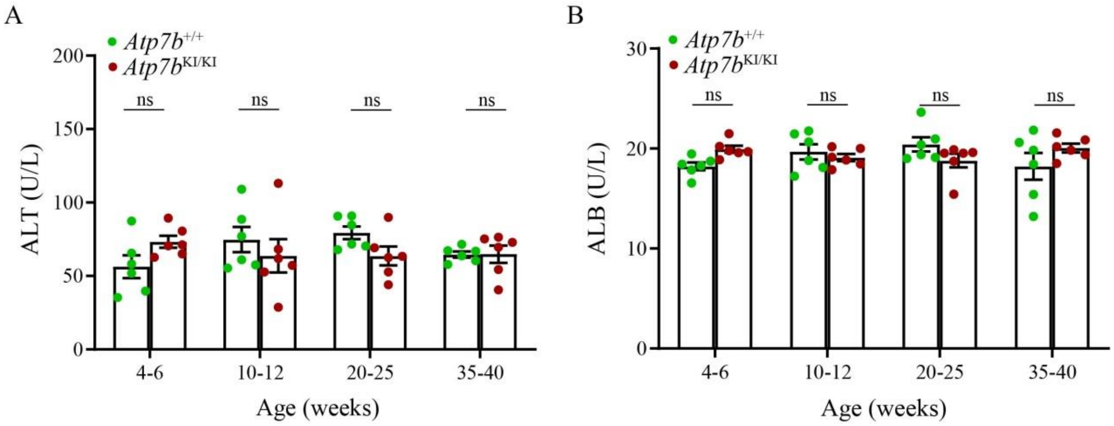

Figure S4. Liver functions of *Atp7b*$^{\mathbf{KI/KI}}$ and *Atp7b*$^{+/+}$ mice

(A) Comparison of serum ALT levels between *Atp7b*$^{KI/KI}$ and *Atp7b*$^{+/+}$ mice. (B) Comparison of serum ALB levels between *Atp7b*$^{KI/KI}$ and *Atp7b*$^{+/+}$ mice.

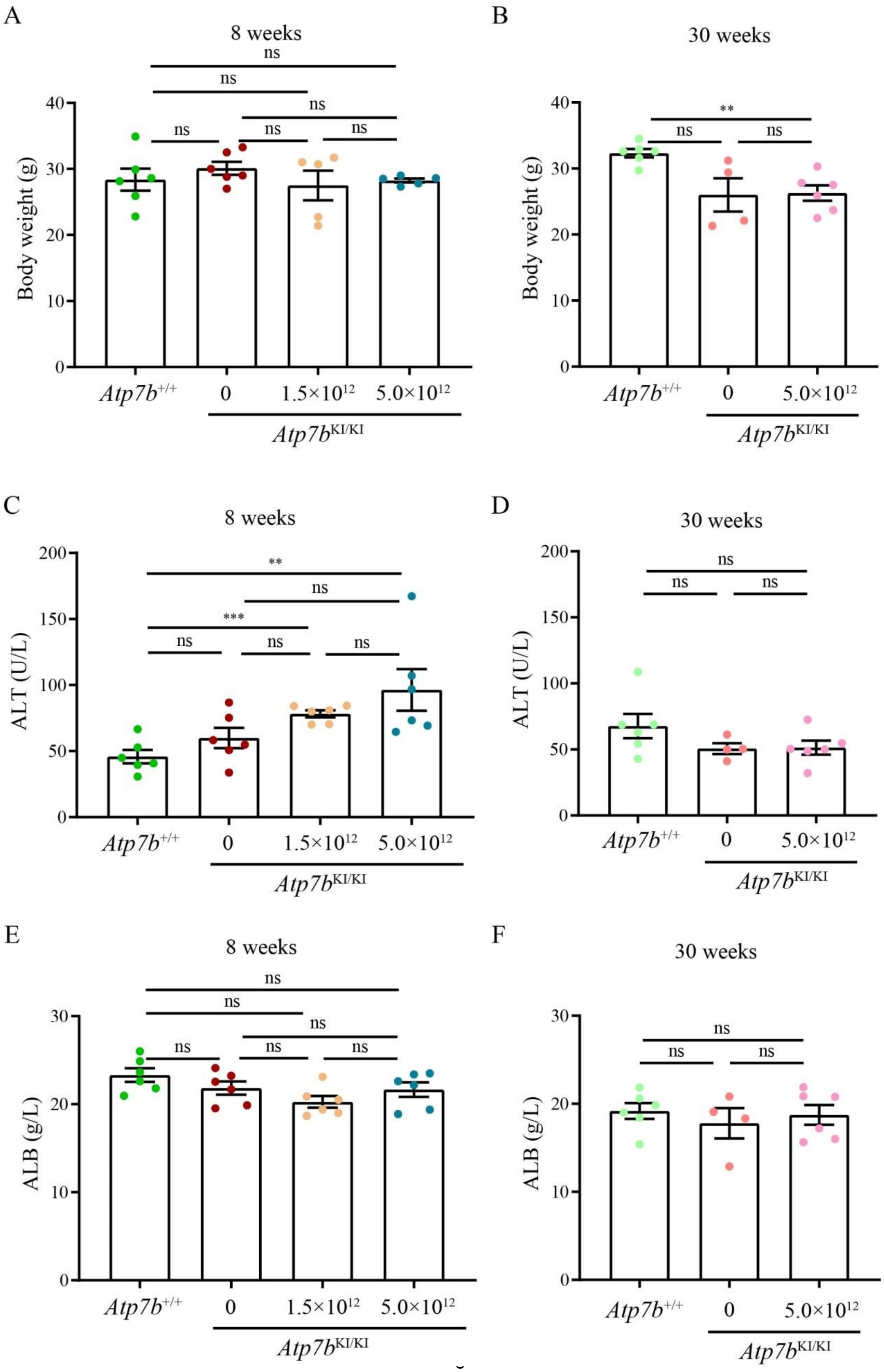
A
8 weeks
Body weight (g)
ns
**
Atp7b+/+
0
1.5×10^12
5.0×10^12
Atp7bKI/KI
B
30 weeks
C
8 weeks
ALT (U/L)
***
D
30 weeks
E
8 weeks
ALB (g/L)
F
30 weeks

Figure S5. The body weight and liver functions in *Atp7b*$^{KI/KI}$ mice after AAV8-Δ C4ATP7B therapy.

(A) Comparison of body weight among treated *Atp7b*$^{KI/KI}$, untreated *Atp7b*$^{KI/KI}$, and *Atp7b*$^{+/+}$ mice at 8 weeks after treatment. (B) Comparison of body weight among treated *Atp7b*$^{KI/KI}$, untreated *Atp7b*$^{KI/KI}$, and *Atp7b*$^{+/+}$ mice at 30 weeks after treatment. (C) Comparison of serum ALT levels among treated *Atp7b*$^{KI/KI}$, untreated *Atp7b*$^{KI/KI}$, and *Atp7b*$^{+/+}$ mice at 8 weeks after treatment. (D) Comparison of serum ALT levels among treated *Atp7b*$^{KI/KI}$, untreated *Atp7b*$^{KI/KI}$, and *Atp7b*$^{+/+}$ mice at 30 weeks after treatment. (E) Comparison of serum ALB levels among treated *Atp7b*$^{KI/KI}$, untreated *Atp7b*$^{KI/KI}$, and *Atp7b*$^{+/+}$ mice at 8 weeks after treatment. (F) Comparison of serum ALB levels among treated *Atp7b*$^{KI/KI}$, untreated *Atp7b*$^{KI/KI}$, and *Atp7b*$^{+/+}$ mice at 30 weeks after treatment.

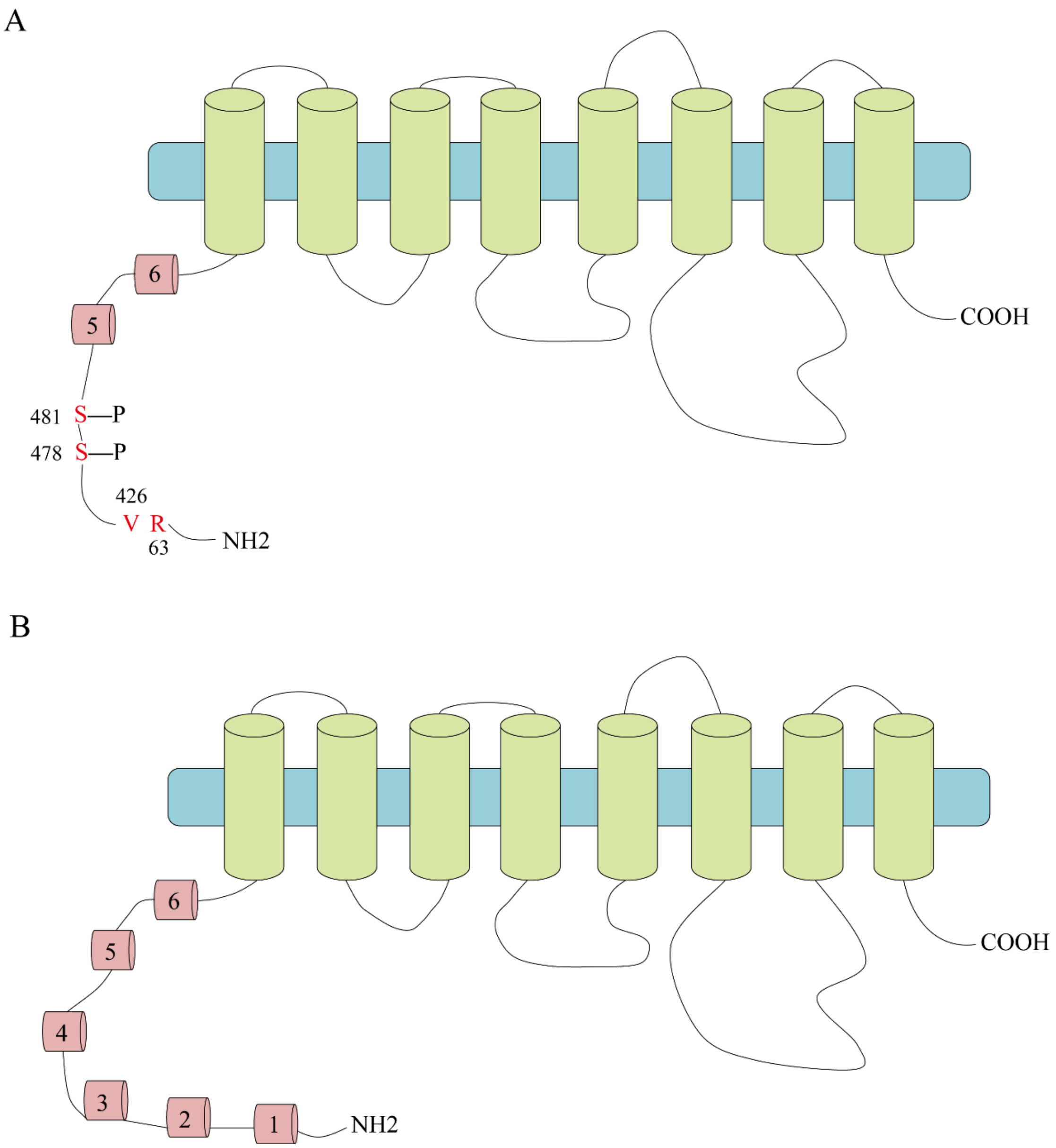

Figure S6. Reconstruction of truncated human ATP7B (ΔC4ATP7B) proteins.

(A) Schematic representation of truncated ATP7B (ΔC4ATP7B) proteins. In ΔC4ATP7B protein, the first 4 amino-terminal metal-binding domains (pink boxes) have been eliminated, 63 amino acids at the N-terminal have been preserved and the sequence containing the ser478 and ser481 phosphorylation sites between MBD4-5 have been retained. (B) Schematic representation of full-length ATP7B proteins.

Table S1. Study design for evaluation of AAV8-ΔC4ATP7B gene therapy.

Group	Genotype	N	Gender	Age (weeks)	Dose of AAV8-ΔC4ATP7B (vg/kg)	Sacrifice time after treatment (weeks)
1 (control)	*Atp7b*$^{+/+}$	6	3M/3F	10	0	8
2 (control)	*Atp7b*$^{KI/KI}$	6	3M/3F	10	0	8
3 (treatment)	*Atp7b*$^{KI/KI}$	6	5M/1F	10	$1.5x10^{12}$	8
4 (treatment)	*Atp7b*$^{KI/KI}$	6	5M/1F	10	$5.0x10^{12}$	8
5 (control)	*Atp7b*$^{+/+}$	6	3M/3F	10	0	30
6 (control)	*Atp7b*$^{KI/KI}$	6	3M/3F	10	0	30
7 (treatment)	*Atp7b*$^{KI/KI}$	6	3M/3F	10	$5.0x10^{12}$	30

N, number; M, male; F, female

Table S2. Primer sequences for qPCR assays.

Gene	Forward primer (5’→3’)	Reverse primer (5’→3’)
GAPDH	AGGTCGGTGTGAACGGATTTG	GGGGTCGTTGATGGCAACA
ΔC4ATP7B	AGAGCTTCGCCTTTGACAAC	TCTCAGACACCACTCTCACG
AAV vector	TACCTTTTTTACAGGGTACCGCCACCATGC	TCAGACACCACTCTCACGGTGGAGGT
