# Supplementary material for: Evaluation of efficacy and safety of AAV8-ΔC4ATP7B gene therapy in a mutant mouse model of Wilson’s disease
Source: Mol Ther Methods Clin Dev. 2025 Feb 13;33(1):101435. doi: 10.1016/j.omtm.2025.101435 (PMC11919453; doi:10.1016/j.omtm.2025.101435)
Supplement: Document S2. Article plus supplemental information [file mmc2.pdf]

Molecular Therapy
Methods & Clinical Development
Original Article

Evaluation of efficacy and safety of AAV8-ΔC4ATP7B gene therapy in a mutant mouse model of Wilson's disease

Chunhua Zeng,[1,7] Yunting Lin,[1,7] Xinshuo Lu,[1,7] Shehong Chen,[1,2,7] Yan Xia,[3] Kangdi Zhang,[1] Yongxian Shao,[1] Zhihong Guan,[1] Rong Du,[1] Zongcai Liu,[1] Mingqi Zhao,[4] Xiaoling Jiang,[5] Yanna Cai,[1] Taolin Li,[1] Xueying Su,[1] Yaoyong Chen,[2] Xiaoyan Dong,[3] Wen Zhang,[1] Li Liu,[1] and Wenhao Zhou[6]

[1]Department of Genetics and Endocrinology, Guangzhou Women and Children's Medical Center, Guangzhou Medical University, Guangdong Provincial Clinical Research Center for Child Health, Guangzhou 510623, China; [2]Department of Pediatrics, The Third Affiliated Hospital of Guangzhou Medical University, Guangzhou 510150, China; [3]GeneCradle Therapeutics, Beijing 100176, China; [4]Center Laboratory, Guangzhou Women and Children's Medical Center, Guangzhou Medical University, Guangdong Provincial Clinical Research Center for Child Health, Guangzhou 510623, China; [5]Children's Hospital, Zhejiang University School of Medicine, National Clinical Research Center for Child Health, Hangzhou 310000, China; [6]Division of Neonatology and Center for Newborn Care, Guangzhou Women and Children's Medical Center, Guangzhou Medical University, Guangdong Provincial Clinical Research Center for Child Health, Guangzhou 510623, China

Wilson's disease (WD) is an autosomal recessive disorder caused by pathogenic variants in the *ATP7B* gene, resulting in the toxic accumulation of copper (Cu). Impaired Cu homeostasis in WD is characterized by low serum ceruloplasmin, excess hepatic Cu, and elevated urinary Cu. WD often presents with hepatic and/or neurological diseases and is fatal if untreated. Adeno-associated virus (AAV)-mediated gene therapy holds promise for WD, but challenges remain in efficacy and safety. Here, we established an *Atp7b* R780L knockin (KI) mouse model corresponding to the human *ATP7B* R778L variant and investigated the therapeutic efficacy and safety of liver-targeted AAV8-mediated *ATP7B* (AAV8-ΔC4ATP7B) gene therapy in this model. The results demonstrated the *Atp7b*$^{KI/KI}$ mice recapitulated key features of impaired Cu metabolism in WD but had mild liver disease. Ten-week-old *Atp7b*$^{KI/KI}$ mice received a single-dose of AAV8-ΔC4ATP7B and were sacrificed at 8 or 30 weeks after treatment. Treated *Atp7b*$^{KI/KI}$ mice showed normalization of serum ceruloplasmin, reduced hepatic Cu, decreased urinary Cu, and reversed liver histopathology. Serum transaminases had a transient increase at 8 weeks after treatment but returned to normal at 30 weeks after treatment. These data provide evidence for the efficacy and safety of AAV8-ΔC4ATP7B in animals, supporting clinical translation to patients with WD.

INTRODUCTION

Wilson's disease (WD; OMIM 277900) is an autosomal recessive disorder caused by pathologic variants in the copper (Cu)-transporting ATPase beta (*ATP7B*) gene.[1–3] The pathologic variants in the *ATP7B* gene result in the deficiency of ATP7B protein, leading to excess accumulation of Cu in the liver, brain, kidney, and other organs. WD is a disorder of Cu metabolism. Impaired Cu homeostasis in WD is characterized by low serum ceruloplasmin, elevated urinary Cu excretion, excess Cu accumulation, liver inflammation, and fibrosis.[4,5] WD can present at any age with a wide range of clinical manifestations, from asymptomatic aminotransferase elevations to severe liver disease such as cirrhosis and liver failure, neurological symptoms, and renal disease. A minority of WD cases present with cardiac disease, endocrine symptoms, and musculoskeletal impairment. WD is fatal if left untreated.[6–8]

The global prevalence of WD is estimated to be 1/20,000–1/10,000, with higher rates in Asian populations than in Western countries. Different from the prevalent H1069Q variant of the *ATP7B* gene in most European countries with an allele frequency range of 15%–72%, the most common variant in most Asian countries is the R778L variant, with an allele frequency range of 13%–56%.[9,10]

A well-characterized animal model is commonly used to elucidate pathological mechanisms and explore therapeutic targets of human genetic diseases.[11–14] To date, several WD animal models, including

Received 16 September 2024; accepted 11 February 2025;
https://doi.org/10.1016/j.omtm.2025.101435.

[7]These authors contributed equally

Correspondence: Xiaoyan Dong, GeneCradle Therapeutics, Beijing 100176, China.
E-mail: dongxiaoyan@bj-genecradle.com
Correspondence: Wen Zhang, Department of Genetics and Endocrinology, Guangzhou Women and Children's Medical Center, Guangzhou Medical University, Guangzhou 510623, China.
E-mail: zhw2001zhw@163.com
Correspondence: Li Liu, Department of Genetics and Endocrinology, Guangzhou Women and Children's Medical Center, Guangzhou Medical University, Guangzhou 510623, China.
E-mail: liliuchina@qq.com
Correspondence: Wenhao Zhou, Division of Neonatology and Center for Newborn Care, Guangzhou Women and Children's Medical Center, Guangzhou Medical University, Guangzhou 510623, China.
E-mail: zhouwenhao@fudan.edu.cn

© 2025 The Authors. Published by Elsevier Inc. on behalf of The American Society of Gene and Cell Therapy.
This is an open access article under the CC BY license (http://creativecommons.org/licenses/by/4.0/).

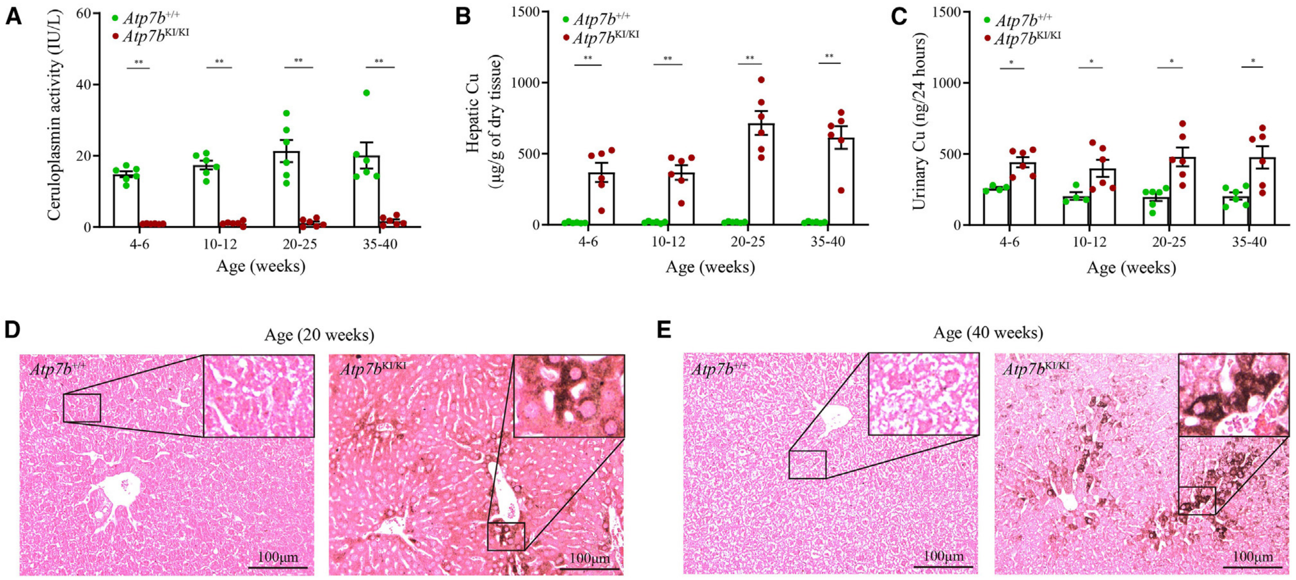

Figure 1. Impaired Cu metabolism and excess hepatic Cu deposition in *Atp7b*$^{KI/KI}$ mice

(A) Comparison of serum ceruloplasmin levels between *Atp7b*$^{KI/KI}$ and *Atp7b*$^{+/+}$ mice. (B) Comparison of hepatic Cu content between *Atp7b*$^{KI/KI}$ and *Atp7b*$^{+/+}$ mice. (C) Comparison of urinary Cu levels between *Atp7b*$^{KI/KI}$ and *Atp7b*$^{+/+}$ mice. The above data are presented as mean ± SEM. *$p < 0.05$; **$p < 0.01$. (D and E) Timm's sulfide silver staining of liver sections in 20- and 40-week-old *Atp7b*$^{KI/KI}$ and *Atp7b*$^{+/+}$ mice. Objective, ×20. Scale bar, 100 μm.

Atp7b knockout (KO) mice, toxic-milk mice, Long-Evans Cinnamon (LEC) rats, and Labrador retrievers, have been reported.[15–18] However, these animal models are not fully representative of the varied clinical presentations and genotypes of human patients with WD. In this study, we established a novel *Atp7b* R780L knockin (KI) mouse model (*Atp7b*$^{KI/KI}$) to mimic the most prevalent pathogenic variant c.2333G>T(p.R778L) in the *ATP7B* gene among Asian patients of WD and further characterized its phenotypes and metabolic profiles.

Current treatment strategies for WD are focused mainly on decreasing Cu absorption with zinc and a low-Cu diet and increasing urinary Cu excretion with Cu-chelating drugs. Liver transplantation is indicated for severe WD cases with liver failure or cirrhosis.[19–25] Although current treatments have shown promising results in most WD patients, they are not effective for all WD patients and have many limitations, such as poor response, intolerance, allergy, and poor compliance. In addition, the long-term complications of current treatment remain a major problem.[26,27] Therefore, alternative and more effective therapies are highly desirable.

In recent years, liver-directed adeno-associated virus (AAV) vector-based gene therapies have shown great potential for the treatment of WD, and several AAV-mediated deliveries of human *ATP7B* (*hATP7B*) into mouse liver have proven the effectiveness in rescuing hepatic Cu homeostasis in WD mouse models.[28–32] However, the full length of the *ATP7B* gene in combination with the promoter and poly(A) signal exceeds the cargo size of an AAV vector and results in low-yield production. Due to the large size of *ATP7B* cDNA, the strategy adopted by previous studies was to create a shorter therapeutic vector to improve the production yields.[29,30] Currently, AAV-based gene therapy for WD faces two main challenges: one is the efficiency and durability of transgene expression in the liver, and another is the risk of immune reactions and liver damage with the introduction of foreign DNA into human hepatocytes.[33–35] In this study, we designed a novel AAV8 vector expressing truncated hATP7B protein (AAV8-ΔC4ATP7B) and further investigated its efficacy and safety for WD.

RESULTS

The *Atp7b*$^{KI/KI}$ mice recapitulated impaired Cu metabolism in WD but only developed mild liver disease

The *Atp7b* R780L KI mouse model was generated to mimic the human *ATP7B* R778L variant of WD (Figures S1A and S1B). The *Atp7b*$^{KI/KI}$ mice presented a physical appearance, weight gain, and life span that was similar to those of *Atp7b*$^{+/+}$ (wild-type control) mice (Figures S2A and S2B). No gender difference was found in Cu metabolism biomarkers of all age groups of *Atp7b*$^{+/+}$ and *Atp7b*$^{KI/KI}$ mice (Figures S2C–S2H). Therefore, genotype and age rather than gender were grouped in subsequent experiments.

Compared with age-matched *Atp7b*$^{+/+}$ mice, *Atp7b*$^{KI/KI}$ mice exhibited extremely low levels of serum ceruloplasmin and increased hepatic Cu content and urinary Cu excretion starting from the age of 4–6 weeks. The peak level of serum ceruloplasmin was 3.4 IU/L in *Atp7b*$^{KI/KI}$ mice, whereas the value was 37.7 IU/L in *Atp7b*$^{+/+}$ mice (Figure 1A). Hepatic Cu ranged from 98.8 to 1,020.9 μg/g dry liver tissue in *Atp7b*$^{KI/KI}$ mice, which was 20 to 40-fold that of *Atp7b*$^{+/+}$ mice (7.6–27.1 μg/g dry liver tissue) (Figure 1B). High Cu

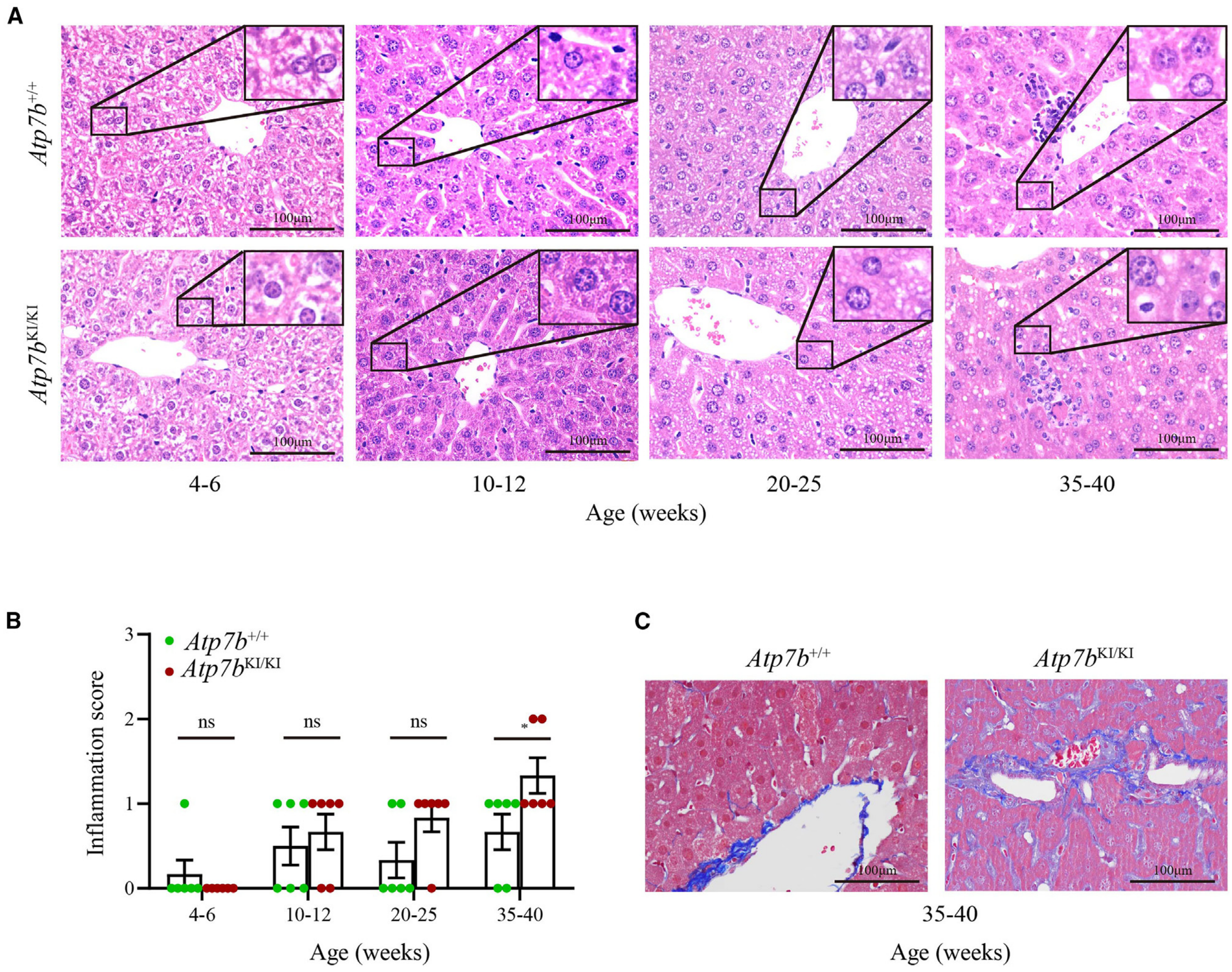

Figure 2. Pathological examination of liver inflammation and fibrosis in *Atp7b*$^{KI/KI}$ mice

(A) H&E staining of livers in *Atp7b*$^{KI/KI}$ and *Atp7b*$^{+/+}$ mice. Objective, ×40. Scale bars, 100 μm. (B) The liver inflammation score of *Atp7b*$^{KI/KI}$ and *Atp7b*$^{+/+}$ mice. The data are presented as mean ± SEM. ns, $p > 0.05$; *$p < 0.05$. (C) Masson staining of livers in *Atp7b*$^{KI/KI}$ and *Atp7b*$^{+/+}$ mice. Objective, ×40. Scale bar, 100 μm.

content was also observed in the kidney and brain of *Atp7b*$^{KI/KI}$ mice at the age of 35–40 weeks (Figures S3A and S3B). Urinary Cu excretion ranged from 227.3 to 714.8 ng/24 h in *Atp7b*$^{KI/KI}$ mice, which was significantly higher than age-matched *Atp7b*$^{+/+}$ mice (Figure 1C). Timm's sulfide silver staining of the livers of *Atp7b*$^{KI/KI}$ mice showed extensive Cu deposition, which was unevenly distributed and age related (Figures 1D and 1E).

In contrast to the severe impairment of Cu homeostasis, *Atp7b*$^{KI/KI}$ mice developed only mild liver disease without neurological damage throughout life. Serum alanine aminotransferase (ALT) and albumin (ALB) levels in *Atp7b*$^{KI/KI}$ mice remained normal at the age of 35–40 weeks (Figure S4). Inflammatory cell infiltration in the livers of *Atp7b*$^{KI/KI}$ mice increased obviously with age (Figure 2A), but the statistical difference between *Atp7b*$^{KI/KI}$ and *Atp7b*$^{+/+}$ mice was not derived until the elder age of 35–40 weeks (Figure 2B). Moreover, Masson staining of the livers of *Atp7b*$^{KI/KI}$ mice showed more severe fibrosis than that of *Atp7b*$^{+/+}$ mice (Figure 2C).

The above results showed that the *Atp7b*$^{KI/KI}$ mice recapitulated key features of impaired Cu metabolism in WD and developed mild liver inflammation, but retained normal liver function, indicating that this *Atp7b*$^{KI/KI}$ mutant mouse model of WD exhibited a mild disease phenotype of WD.

Transgene expression distributed predominately in the livers of AAV8-ΔC4ATP7B-treated mice

To achieve efficient and liver-targeted expression of the *hATP7B* gene, we designed a novel AAV construct by focusing on two aspects: first, a short liver-specific promoter and an artificially designed poly(A) tailing signal were selected to control the length of the ITR-promoter-ATP7B-polyA-ITR sequence within 5.1 kb. Second,

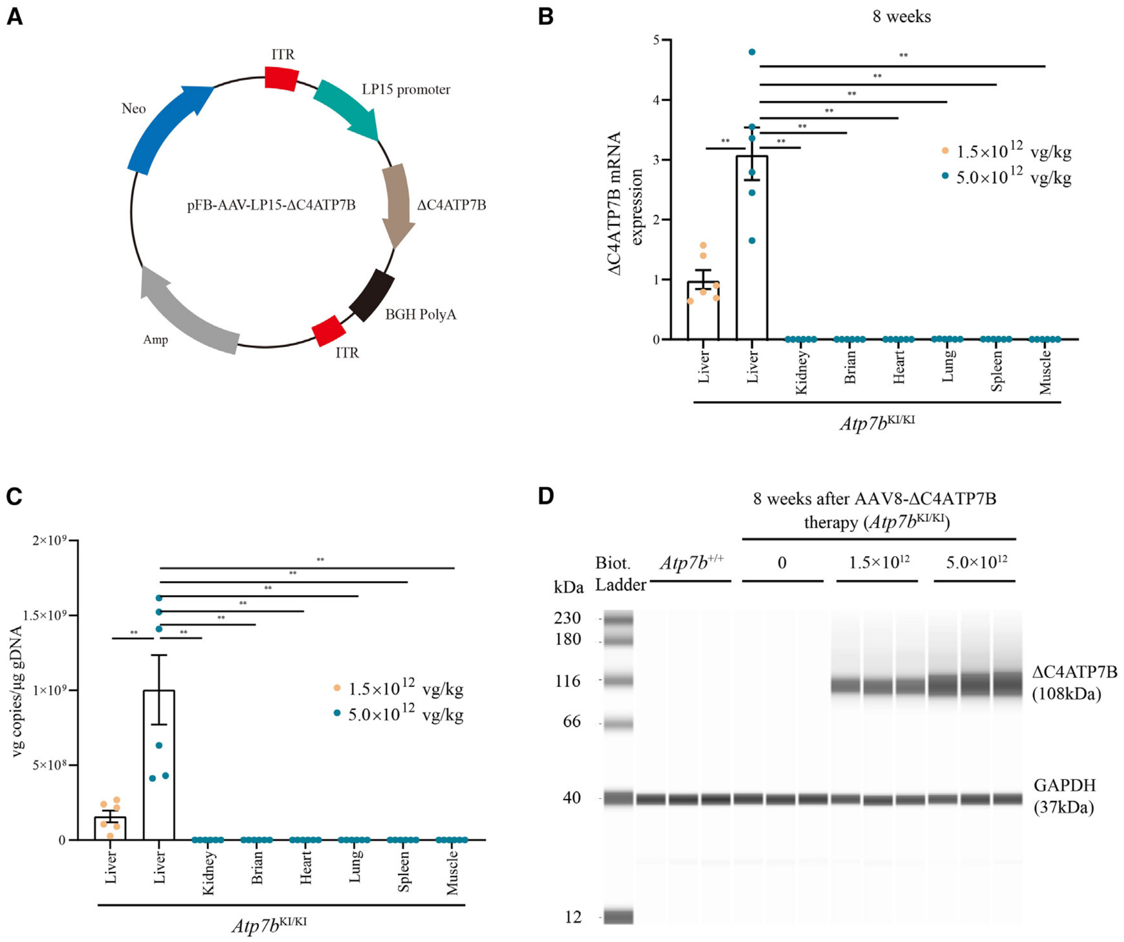

Figure 3. Construction of the AAV8-ΔC4ATP7B vector and measurement of its transgene expression in *Atp7b*$^{KI/KI}$ mice

(A) Schematic representation of the genome components of the AAV8-ΔC4ATP7B vector. (B) qPCR results of *ΔC4ATP7B* expression in different organs of *Atp7b*$^{KI/KI}$ mice 8 weeks after treatment. (C) qPCR results of AAV8 vector genome copy numbers in different organs of *Atp7b*$^{KI/KI}$ mice 8 weeks after treatment. qPCR results are presented as mean ± SEM; *$p < 0.05$; **$p < 0.01$. (D) Western blot analysis of ΔC4ATP7B protein in liver lysates 8 weeks after treatment.

the first four of the six Cu ion binding sequences in the ATP7B protein sequence were deleted, with the reserve of 63 amino acids at the N-terminal retained according to a previous study.[36] With this strategy, an artificial cDNA encoding codon-optimized truncated hATP7B protein (less than 1,100 amino acids), called ΔC4ATP7B, was generated. The plasmid pFB-AAV8-LP15-ΔC4ATP7B was constructed for recombinant AAV packaging (AAV8-ΔC4ATP7B), which enables the expression of *hATP7B* under the control of a liver-specific promoter (Figure 3A).

To investigate whether the administration of AAV8-ΔC4ATP7B could result in liver-targeted transgene expression and restore impaired Cu homeostasis, a low dose (1.5×10^{12} vector genomes [vg]/kg) and a high dose (5.0×10^{12} vg/kg) of AAV8-ΔC4ATP7B were injected into mice of two therapeutic groups at the age of 10 weeks. Quantitative polymerase chain reaction (qPCR) confirmed the dose-dependent and liver-specific expression of *ΔC4ATP7B* (Figure 3B). AAV vector genome copy numbers at 8 weeks after treatment were also confined to the liver rather than the brain, kidney, heart, lung, spleen, and muscle (Figure 3C). Western blot further confirmed that the expression of ΔC4ATP7B protein at 8 weeks after treatment was dose dependent in treated *Atp7b*$^{KI/KI}$ mice (Figure 3D).

AAV8-ΔC4ATP7B therapy rescued impaired Cu metabolism and reversed liver histopathology of *Atp7b*$^{KI/KI}$ mice

To assess the efficacy of AAV8-ΔC4ATP7B therapy, 10-week-old *Atp7b*$^{KI/KI}$ mice were divided into two therapeutic groups by receiving a low dose (1.5×10^{12} vg/kg) and a high dose (5.0×10^{12} vg/kg) of AAV8-ΔC4ATP7B. Age-matched *Atp7b*$^{KI/KI}$ mice without treatment served as disease controls, while age-matched *Atp7b*$^{+/+}$ mice served as healthy controls. Key parameters of impaired Cu metabolism and hepatic histopathology in *Atp7b*$^{KI/KI}$ mice after injection of AAV8-ΔC4ATP7B were detected (Table S1).

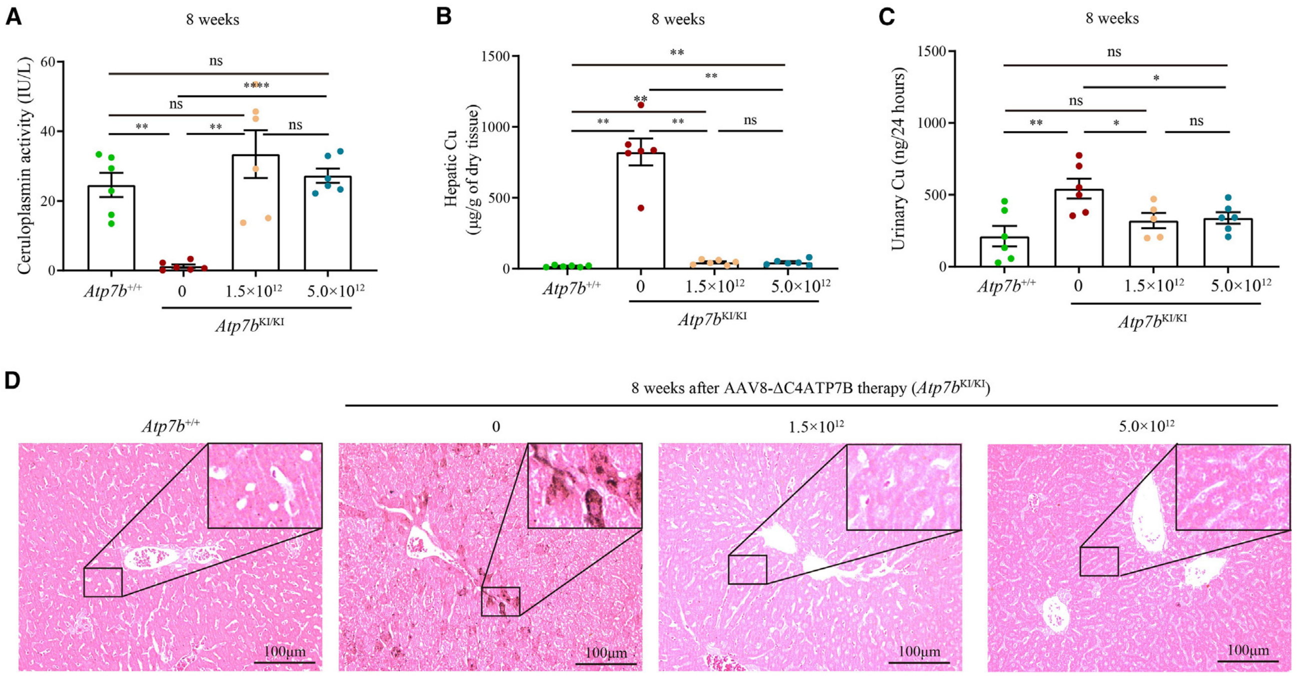

Figure 4. AAV8-ΔC4ATP7B therapy rescued impaired Cu metabolism and reduced hepatic Cu deposition in *Atp7b*$^{KI/KI}$ mice 8 weeks after treatment
(A) Comparison of serum ceruloplasmin levels among treated *Atp7b*$^{KI/KI}$, untreated *Atp7b*$^{KI/KI}$, and *Atp7b*$^{+/+}$ mice. (B) Comparison of hepatic Cu content among treated *Atp7b*$^{KI/KI}$, untreated *Atp7b*$^{KI/KI}$, and *Atp7b*$^{+/+}$ mice. (C) Comparison of urinary Cu levels among treated *Atp7b*$^{KI/KI}$, untreated *Atp7b*$^{KI/KI}$, and *Atp7b*$^{+/+}$ mice. The above data are presented as mean ± SEM. ns, $p > 0.05$; *$p < 0.05$; **$p < 0.01$; ****$p < 0.0001$. (D) Timm's sulfide silver staining of liver sections in treated *Atp7b*$^{KI/KI}$, untreated *Atp7b*$^{KI/KI}$, and *Atp7b*$^{+/+}$ mice. Objective, ×20. Scale bar, 100 μm.

Eight weeks after the administration of one single dose of AAV8-ΔC4ATP7B, serum ceruloplasmin was normalized (Figure 4A) and liver Cu content, urinary Cu excretion and hepatic Cu deposition were significantly reduced (Figures 4B–4D), but kidney and brain Cu content, inflammatory cell infiltration, and Masson staining of the livers resulted in no significant change in treated *Atp7b*$^{KI/KI}$ mice compared with untreated *Atp7b*$^{KI/KI}$ mice (Figures 4B–4D, 5A, 5B, and 6).

To evaluate the long-term outcome of AAV8-ΔC4ATP7B therapy, 10-week-old *Atp7b*$^{KI/KI}$ mice were treated with a single high dose (5.0×10^{12} vg/kg) of AAV8-ΔC4ATP7B and sacrificed at 30 weeks after treatment. Similarly, treated *Atp7b*$^{KI/KI}$ mice exhibited normalized serum ceruloplasmin, decreased hepatic Cu content, reduced urinary Cu excretion, and reduced hepatic Cu deposition (Figure 7). Unlike the short-term (8-week) results after treatment, inflammatory cell infiltration and liver fibrosis in treated *Atp7b*$^{KI/KI}$ mice were less than those in untreated mice at 30 weeks after treatment (Figure 8). In addition, the Cu levels in the kidneys of treated *Atp7b*$^{KI/KI}$ mice were reduced and significantly lower than those of untreated mice, whereas Cu levels in the brains of treated *Atp7b*$^{KI/KI}$ mice remained unchanged (Figures 5C and 5D).

These results indicated that AAV8-ΔC4ATP7B gene therapy resulted in complete and sustained normalization of serum ceruloplasmin, reduced hepatic Cu accumulation, decreased urinary Cu excretion, and reversal of liver histopathology in the treated *Atp7b*$^{KI/KI}$ mouse model.

AAV8-ΔC4ATP7B showed long-term safety in treated Atp7b$^{KI/KI}$ mice

To investigate the safety of AAV8-ΔC4ATP7B therapy, we analyzed parameters such as body weight, serum ALT and serum ALB of treated *Atp7b*$^{KI/KI}$ mice at 8 weeks and 30 weeks after administration of AAV8-ΔC4ATP7B. The gross appearance and weight gain of *Atp7b*$^{KI/KI}$ mice treated with AAV8-ΔC4ATP7B were similar in untreated *Atp7b*$^{KI/KI}$ mice and *Atp7b*$^{+/+}$ control mice (Figures S5A and S5B). The serum ALT levels of *Atp7b*$^{KI/KI}$ mice had a transient increase at 8 weeks after treatment (Figure S5C) and returned to the normal range (10.06–96.47 U/L) at 30 weeks after treatment (Figure S5D). All treated *Atp7b*$^{KI/KI}$ mice maintained serum ALB levels within the normal range throughout the experimental period (Figures S5E and S5F).

As AAV-mediated gene therapy has the potential to cause hepatocellular carcinoma, hematoxylin and eosin (H&E) staining was performed to investigate the livers of *Atp7b*$^{KI/KI}$ mice at 30 weeks after the administration of AAV8-ΔC4ATP7B, and no sign of abnormal morphology or pleomorphism in the livers of treated *Atp7b*$^{KI/KI}$ mice was found (Figure 8).

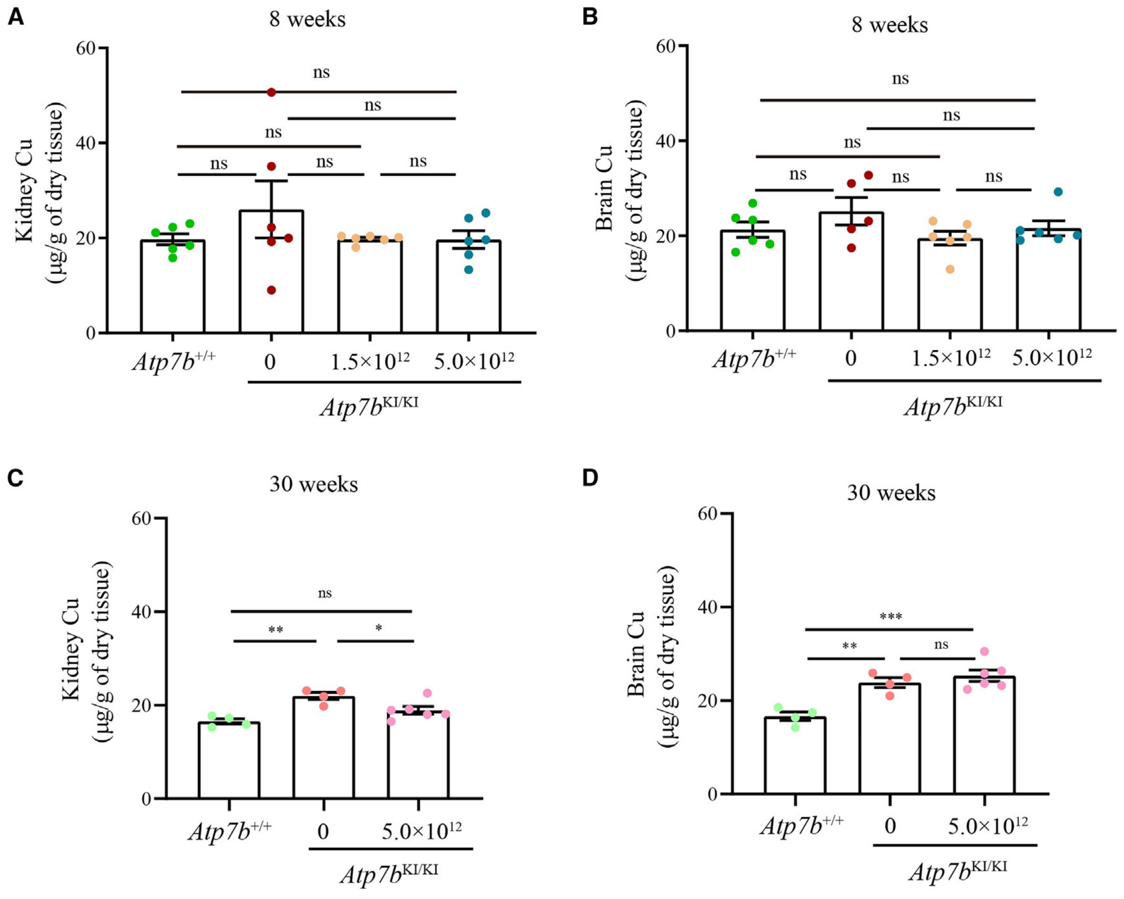

Figure 5. Analysis of Cu content in kidney and brain of *Atp7b*$^{KI/KI}$ mice after AAV8-ΔC4ATP7B treatment
(A) Comparison of kidney Cu content among treated *Atp7b*$^{KI/KI}$, untreated *Atp7b*$^{KI/KI}$, and *Atp7b*$^{+/+}$ mice 8 weeks after treatment. (B) Comparison of brain Cu content among treated *Atp7b*$^{KI/KI}$, untreated *Atp7b*$^{KI/KI}$, and *Atp7b*$^{+/+}$ mice 8 weeks after treatment. (C) Comparison of kidney Cu content among treated *Atp7b*$^{KI/KI}$, untreated *Atp7b*$^{KI/KI}$, and *Atp7b*$^{+/+}$ mice 30 weeks after treatment. (D) Comparison of brain Cu content among treated *Atp7b*$^{KI/KI}$, untreated *Atp7b*$^{KI/KI}$, and *Atp7b*$^{+/+}$ mice 30 weeks after treatment. The above data are presented as mean ± SEM. ns, $p > 0.05$; *$p < 0.05$; **$p < 0.01$; ***$p < 0.001$.

DISCUSSION

The currently available treatments for WD require lifelong compliance with the medication and low-Cu diet. Medication compliance and pharmacological side effects limit the long-term prognosis of WD patients. Gene therapy with recombinant AAV holds excellent potential for inherited metabolic diseases.[37,38] The concept of AAV gene therapy for WD has been reported by several research groups.[28–30] Their results indicate that this conceptual approach is applicable. To date, two gene therapies for WD are undergoing clinical trials (these trials were registered at ClinicalTrials.gov: NCT04537377 and NCT04884815). However, some challenges are hampering the therapeutic efficacy of AAV for WD treatment.

One challenge is the efficiency of hepatocytes-targeted transgene delivery and expression. In this study, the AAV vector construct is different from those previous studies. As we know, the full length of the ATP7B protein of 1,465 amino acids surpasses the optimal packaging capacity of recombinant AAV vector, whereas deletion of MBD1-4 does not affect Cu induction and excretion.[36] Therefore, we designed a codon-optimized sequence that removes MBD1-4 from the ATP7B protein and preserves 63 amino acids at the N-terminal. Moreover, we constructed an AAV vector containing ΔC4ATP7B cDNA and a liver-specific promoter (LP15). Compared with previous studies, our ΔC4ATP7B had a better therapeutic effect at the same dose (1.5×10^{12} vg/kg) of AAV8 vector,[29] which rapidly normalized serum ceruloplasmin and reduced liver Cu content and urinary Cu excretion. Both short-term (8 weeks) and long-term (30 weeks) outcomes after treatment with AAV8-ΔC4ATP7B in *Atp7b*$^{KI/KI}$ mice exhibited normalization of serum ceruloplasmin and significantly decreased hepatic Cu, indicating that AAV8-ΔC4ATP7B achieved a persistent expression of *hATP7B* and a sustainable therapeutic efficacy to rescue Cu homeostasis of the *Atp7b* KI mouse model.

Another challenge is the risk of AAV-related immune reactions.[33–35,39] Notably, safety considerations when using AAVs become more critical due to death reports caused by high-dose AAV gene therapy in clinical

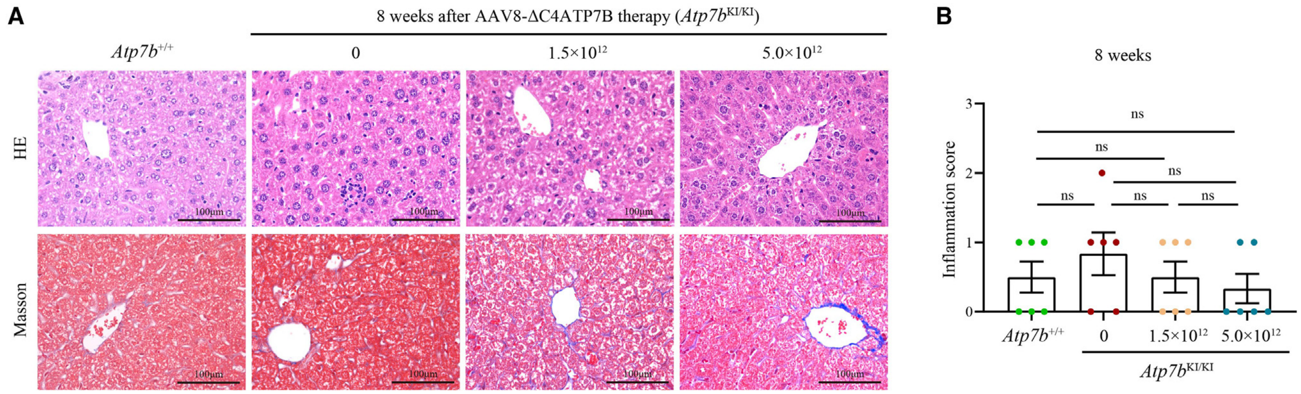

Figure 6. Analysis of liver inflammation in *Atp7b*$^{KI/KI}$ mice 8 weeks after AAV8-ΔC4ATP7B therapy

(A) H&E staining of livers in *Atp7b*$^{KI/KI}$ mice after AAV8-ΔC4ATP7B therapy for 8 weeks. Objective, ×40. Scale bar, 100 μm. (B) The liver inflammation score in *Atp7b*$^{KI/KI}$ mice after 8 weeks of treatment. The data are presented as mean ± SEM. ns, $p > 0.05$.

trials.[40] A common side effect of AAV-mediated gene therapy is AAV-related liver damage with elevated ALT levels, which may exacerbate the liver disease of WD patients.[33,39] Thus, in addition to the capacity to restore Cu homeostasis, the optimal gene medicine for WD should have high transgene expression efficiency and low risk of liver toxicity. As discussed above, the same dose (1.5×10^{12} vg/kg) of AAV8 vector showed a more significant therapeutic effect than in previous studies, indicating fewer concerns about long-term safety. Even though serum ALT levels of treated *Atp7b*$^{KI/KI}$ mice were elevated at 8 weeks after treatment, they returned to within the normal range at 30 weeks after treatment (Figure S5), indicating that the *Atp7b*$^{KI/KI}$ mice treated with ΔC4ATP7B may experience transient elevations of serum ALTs, and the potential hepatotoxicity needs to be addressed in future studies.

Due to the limitations of AAV-based gene therapy, non-viral delivery vehicles have been studied for gene therapies. Lipid nanoparticles (LNPs) are nanoparticles with a phospholipid layer and a lipophilic core for transporting drugs.[10] LNPs, the US Food and Drug Administration-approved formulations targeting the liver, are capable of delivering genetic materials into non-dividing cells with lower immunogenicity than viral vectors. Furthermore, LNPs are non-toxic and user-friendly. In particular, LNPs have an advantage in the encapsulation of larger mRNA or DNA for therapeutic use compared with AAV.[41,42] However, there are also some limitations of LNPs, including difficulty in biological degradation, which may trigger potential hepatotoxicity. Although LNP-based therapy has been proved to be effective for liver disease in multiple studies, LNPs have not yet been applied to WD. Future studies could use LNPs to deliver whole human *ATP7B* to rescue impaired Cu homeostasis in WD.

The c.2333G>T(p.R778L) variant in the *ATP7B* gene is the most prevalent pathogenic variant among Asian patients with WD, and those patients carrying this variant predominantly present with early liver disease and low serum ceruloplasmin.[43–45] In this study, we established an *Atp7b* KI mouse model carrying the *Atp7b*^{R780L} variant corresponding to the most frequent *ATP7B*^{R778L} variant in human WD patients. The homozygous *Atp7b*$^{KI/KI}$ mice had significantly decreased serum ceruloplasmin, severe hepatic Cu accumulation, and increased urinary Cu excretion compared with wild-type control mice, which recapitulates the key features of Cu disorder observed in human WD patients successfully. Different from previously reported animal models, our newly established *Atp7b* KI mouse model presented with a normal ALT level and mild pathological changes in liver tissue. With the consideration of hepatic toxicity potentially caused by gene therapy, normal ALT in the WD animal model may be an advantage to assess the drug-related side effects after gene therapy.

Mice are not an excellent experimental model to evaluate liver-targeted toxicity and immunogenicity of AAV-based gene therapy. However, other animal models of WD, including LEC rats and Labrador retrievers, often develop severe liver disease early and die soon after, suggesting that they are not suitable for preclinical studies.[15] Therefore, our newly established *Atp7b* KI mouse model provides an optimal tool to explore pathological mechanisms of Cu homeostasis and monitor therapeutic responses and drug-related liver disease in pilot studies for WD.

There are some limitations in this study. The numbers of *Atp7b*$^{KI/KI}$ mice that received AAV8-ΔC4ATP7B gene therapy and the follow-up time to evaluate its efficacy and safety of gene therapy are limited. Further animal experimental studies and clinical trials are expected in the future.

In conclusion, our newly established *Atp7b* KI mouse model presents with Cu metabolic disorder of WD. In this mouse model, AAV8-ΔC4ATP7B shows great potential as a novel therapeutic gene medicine for WD and may serve as a safer and longer-lasting therapeutic tool to rescue Cu homeostasis.

MATERIALS AND METHODS

Animals

Heterozygous KI mice (*Atp7b*$^{KI/+}$) were intercrossed to generate homozygous KI (*Atp7b*$^{KI/KI}$) and wild-type (*Atp7b*$^{+/+}$) offspring. A total

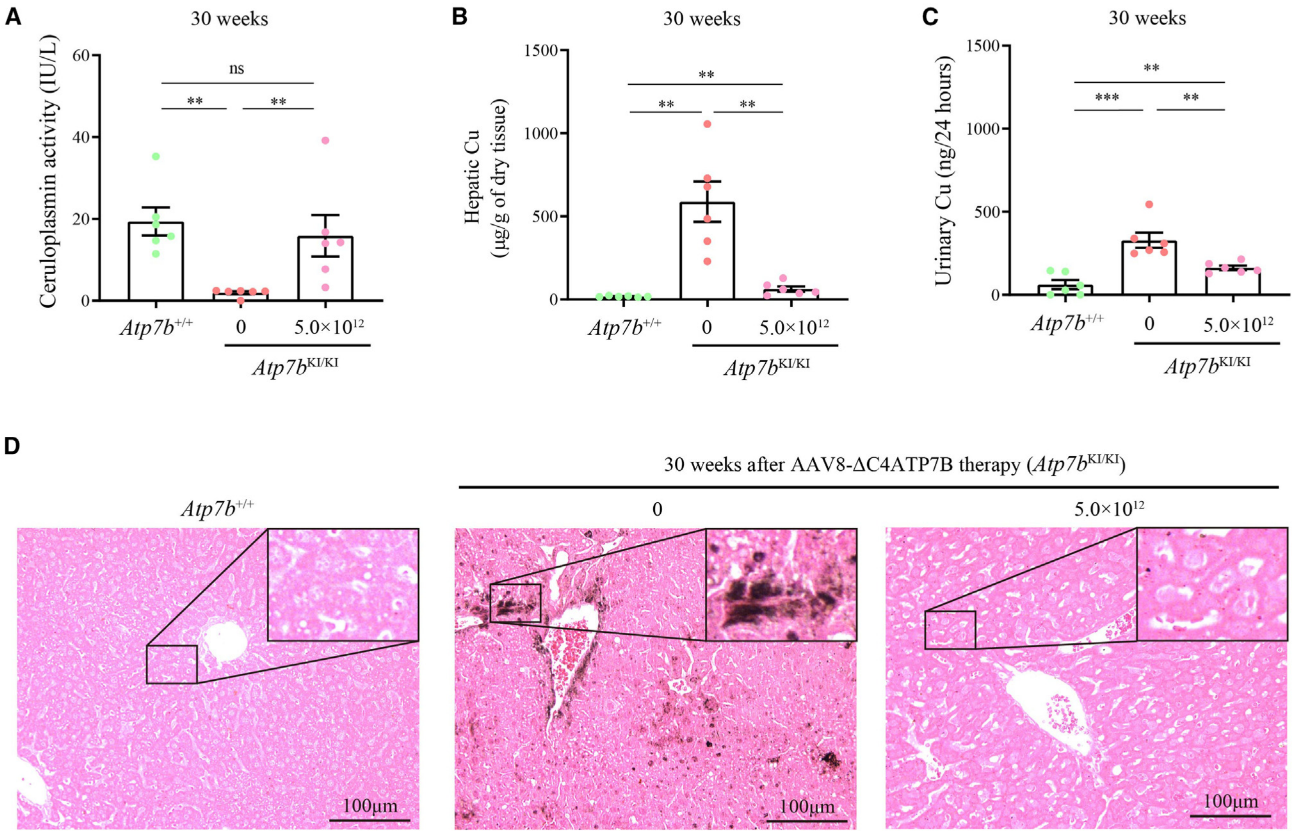

Figure 7. Long-term outcome of AAV8-ΔC4ATP7B therapy in *Atp7b*$^{KI/KI}$ mice 30 weeks after treatment

(A) Comparison of serum ceruloplasmin levels among treated *Atp7b*$^{KI/KI}$, untreated *Atp7b*$^{KI/KI}$, and *Atp7b*$^{+/+}$ mice. (B) Comparison of hepatic Cu content among treated *Atp7b*$^{KI/KI}$, untreated *Atp7b*$^{KI/KI}$, and *Atp7b*$^{+/+}$ mice. (C) Comparison of urinary Cu levels among treated *Atp7b*$^{KI/KI}$, untreated *Atp7b*$^{KI/KI}$, and *Atp7b*$^{+/+}$ mice. The above data are presented as mean ± SEM. ns, $p > 0.05$; $^{**}p < 0.01$; $^{***}p < 0.001$. (D) Timm's sulfide silver staining of liver sections in treated *Atp7b*$^{KI/KI}$, untreated *Atp7b*$^{KI/KI}$, and *Atp7b*$^{+/+}$ mice. Objective, ×20. Scale bar, 100 μm.

of 24 *Atp7b*$^{KI/KI}$ mice were used to characterize the gross appearance, body weight, Cu metabolism, liver function, liver pathology, and behaviors at the ages of 4–6 weeks, 10–12 weeks, 20–25 weeks, and 35–40 weeks. The following primers were used for the genotyping PCR: mAtp7b (R780L) forward: 5′-CAAGCCTACAAATCGCTGAGACA-3′ and mAtp7b (R780L) reverse: 5′-CAGAAGGAACAATCAACAGGGAGA-3′.

All mice were housed in a specific pathogen-free animal facility with a 12-h light/dark schedule. Food and water were provided *ad libitum*. All mouse husbandry and experimental protocols were reviewed and approved by the Institutional Animal Care and Use Committee of Guangzhou Medical University (no. 2019-388).

AAV vector construct

The full length of the human ATP7B protein is 1,465 amino acids, which surpasses the optimal packaging capacity of the recombinant AAV vector. Studies have shown that the deletion of MBD1-4 does not affect Cu induction and excretion, while N-terminal short peptide and MBD5-6 are necessary for protein function.[36] Therefore, we designed a codon-optimized sequence that removes MBD1-4 from the ATP7B coding sequence and preserves 63 amino acids at the N-terminal, which can be translated into ΔC4ATP7B protein (Δ64-425). Then, we constructed an AAV vector containing the ΔC4ATP7B B cDNA and a liver-specific promoter (LP15). A schematic diagram of the construct is shown in Figure S6.

Production of recombinant AAV vector

AAV8-ΔC4ATP7B was produced by Beijing FivePlus Molecular Medicine Institute (China) using the triple plasmid transfection method. Briefly, the helper plasmid pADHelper, Rep/Cap plasmid, and transgene plasmid were mixed at a ratio of 1:1:1. HEK293 cells were transfected with the mixture of the three plasmids, and the cells and culture medium were harvested after 48 h of transfection and purified by ion-exchange chromatography. The viral preparations were desalted and concentrated using an ultrafiltration tube (100 kDa, PALL). The vectors were aliquoted and stored at −80°C. The purity of the vectors was analyzed by SDS-PAGE, and the titers were detected by dot blot assay. The

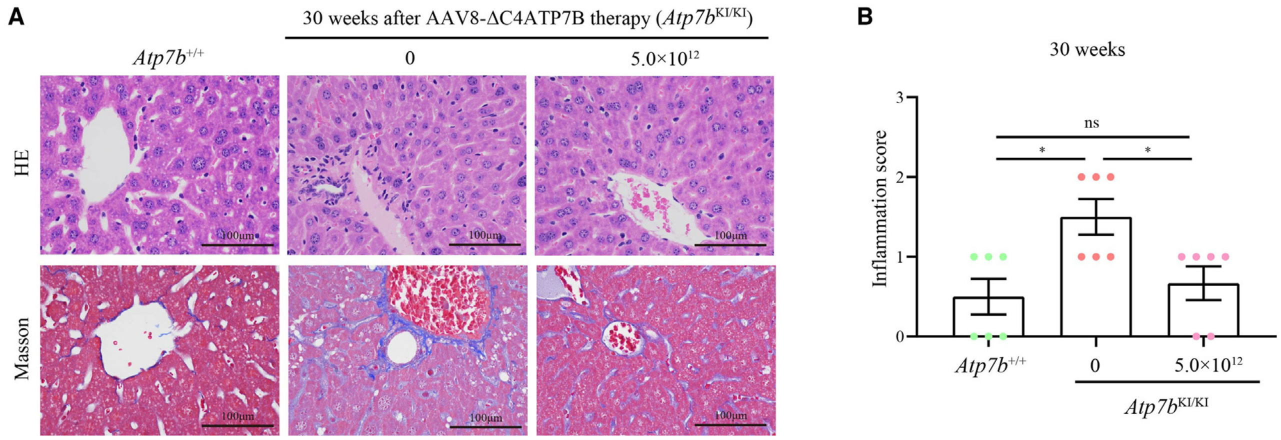

Figure 8. Analysis of liver inflammation in *Atp7b*$^{KI/KI}$ mice 30 weeks after AAV8-ΔC4ATP7B therapy

(A) H&E staining of livers in *Atp7b*$^{KI/KI}$ mice. Objective, ×40. Scale bar, 100 μm. (B) The liver inflammation score of *Atp7b*$^{KI/KI}$ mice. The data are presented as mean ± SEM. ns, $p > 0.05$; $^*p < 0.05$.

recombinant AAV vector genome copy number was detected by qPCR analysis, and the primer sequences used for qPCR are listed in Table S2.

Generation of a C57BL/6 mouse model with *Atp7b*^{R780L} variant

Corresponding to the c.2333G>T(p.R778L) variant in the human *ATP7B* gene (NM_000053.4), the c.2339G>T(p.R780L) variant in the mouse *Atp7b* gene (NM_007511.3) was edited using the CRISPR-Cas9-mediated genome engineering technique (Figure S1A). The Cas9 mRNA, single-guide RNA (sgRNA), and donor oligo were co-injected into zygotes of the C57BL/6N strain by Cyagen Biosciences (China). The sequence of sgRNA was 5′-CATCGCCCTGGGA CGGTGGCTGG-3′, and the donor oligonucleotide was 5′- AAGAG CCCCGTGACCTTCTTTGACACGCCCCCCATGCTCTTTGTGT TCATCGCCCTGGGACTGTGGCTGGAACACGTGGCCAAGGT AGCTACAGCTTCAAGGGTTCTTTCGTTCTTTTGGTAA-3′.

Two founder KI mice born from microinjected embryos were genotyped and confirmed to be carriers of the heterozygous c.2339G>T(p.R780L) variant in the *Atp7b* gene (Figure S1B). The founder KI mice were further backcrossed with C57BL/6N mice for more than two generations before the formal animal experiments.

Blood, urine, and tissue collection

Blood was collected from the retrobulbar venous plexus for the detection of serum ALT, ALB, and ceruloplasmin. A 24-h urine sample was collected using a mouse metabolic cage for the analysis of urinary Cu excretion.

For tissue collection, littermates were anesthetized and sacrificed via an intraperitoneal injection of pentobarbital. The tissues used for histological analysis were immersed in 4% paraformaldehyde in PBS at 4°C overnight and then processed to obtain paraffin sections according to standard histological procedures. Fresh tissues for Cu quantification were dried in a vacuum centrifugal concentrator and then processed for Cu measurements. The tissues used for other purposes were snap-frozen in liquid nitrogen and stored at −80°C.

Biochemical analysis

Serum ALT and ALB were measured using Wuhan ServiceBio Technology (China) with an automatic biochemical analyzer (Rayto Chemray 800, China). Serum ceruloplasmin was detected by amine colorimetry using a ceruloplasmin detection kit (TE0321, Leagene Biotechnology, China).

Cu quantification

To quantify Cu in tissue, fresh liver, brain, and kidney tissues were dried to approximately 5 mg dry tissue before digestion with 2 mL nitric acid and diluted 10 times with ultrapure water according to a published protocol.[46] Dried tissue was digested and centrifuged to collect the supernatant. For quantification of Cu in urine, a 24-h urine sample was collected, and the volume was recorded.

Cu concentrations in the tissue supernatant and urine sample were detected at the Guangzhou DaAn Clinical Laboratory Center (China) using flame atomic absorption spectrometry (HITACHI Z-2000, Japan). The measuring units of Cu measured in tissue and urine were μg/g dry tissue and ng/24 h, respectively.

AAV8-ΔC4ATP7B treatment

A total of 30 *Atp7b*$^{KI/KI}$ mice were used to explore the efficacy and safety of AAV8-ΔC4ATP7B, 12 of which were used as controls (Table S1). Administration of AAV8-ΔC4ATP7B was performed by intravenous injection via the caudal vein in *Atp7b*$^{KI/KI}$ mice at the age of 10 weeks. Six *Atp7b*$^{KI/KI}$ mice were treated with a low dose of 1.5×10^{12} vg/kg AAV8-ΔC4ATP7B vector, and six *Atp7b*$^{KI/KI}$ mice were treated with a high dose of 5.0×10^{12} vg/kg AAV8-ΔC4ATP7B vector; all 12 mice were sacrificed at 8 weeks after

treatment. Another six *Atp7b*$^{KI/KI}$ mice receiving the high dose of 5.0×10^{12} vg/kg AAV8-ΔC4ATP7B were sacrificed at 30 weeks after treatment. A total of 12 age-matched *Atp7b*$^{KI/KI}$ mice were injected with normal saline via the caudal vein as untreated *Atp7b*$^{KI/KI}$ mice controls. A total of 12 age-matched *Atp7b*$^{+/+}$ mice were used as normal controls. The appearance, weight, Cu metabolism, liver function, liver pathology, and behaviors of littermates were observed or measured.

qPCR analysis of *ΔC4ATP7B* expression

Total RNA was extracted from liver or other organ tissues using TRIzol reagent (Life Technologies, USA), and was reverse transcribed into single-strand cDNA using a PrimeScript RT Master Mix kit (Takara, Japan). All procedures were performed in accordance with the manufacturer's protocol. qPCR was performed using a 7500 FAST Real-Time PCR system (Applied Biosystems, USA). All samples were measured in triplicate and quantified using the comparative cycle threshold method for relative gene expression, with normalization to the reference *GAPDH* gene. The primer sequences used for qPCR are listed in Table S2.

Western blot analysis of ΔC4ATP7B protein

Liver tissues were homogenized in radioimmunoprecipitation assay buffer (ServiceBio) containing a protease inhibitor cocktail (Selleckchem, USA). Western blot analysis was performed to identify ΔC4ATP7B protein according to the standard protocol using an antibody against human ATP7B (1:3,000, no. ab124973, Abcam, UK). GAPDH (1:200, no. AF7021, Affinity BioSciences, USA) was used as the reference. The bands were visualized using the Protein Simple whole exome sequencing immunoassay (Protein Simple, USA).

Liver pathology and Cu staining

Standard H&E staining was performed to examine liver morphology and inflammation. To assess hepatic histopathology of *Atp7b*$^{KI/KI}$ mice, we used the hepatic inflammation score to evaluate inflammatory cell infiltration in the liver. A 0–3 scoring system was used to determine inflammation of the liver tissue according to the histopathological scoring protocol.[30] Masson staining was performed to detect liver fibrosis.

Timm's sulfide silver staining was used to visualize Cu deposition in liver tissues.[47,48] Timm's staining was performed in *Atp7b*$^{KI/KI}$ mice treated with different doses of AAV8-ΔC4ATP7B and age-matched untreated *Atp7b*$^{KI/KI}$ and *Atp7b*$^{+/+}$ mice.

Statistical analysis

Statistical analyses were performed using GraphPad Prism 8.0.2 (GraphPad Software, USA). Comparisons were made using Student's t test or one-way ANOVA for parametric tests and using the Mann-Whitney *U* test or the Kruskal-Wallis *H* test for nonparametric tests where appropriate. Data were presented as mean ± standard error of the mean (SEM) in the column. Statistical significance was defined as $p < 0.05$; ns > 0.05, $^{*}p < 0.05$, $^{**}p < 0.01$, $^{***}p < 0.001$, and $^{****}p < 0.0001$.

DATA AVAILABILITY

All relevant data supporting the key findings of this study are available within the article and its supplemental information files or from the corresponding author upon reasonable request.

ACKNOWLEDGMENTS

This work was supported by the National Key Research and Development Program of China (2023YFC2706301) and the Joint Research Fund of Guangdong Basic and Applied Basic Research Commission (2021A1515220168).

AUTHOR CONTRIBUTIONS

C.Z., Y.L., X.L., and S.C. performed the experiments, analyzed the data, and drafted the manuscript. Y.C., Y.X., and Y.S. performed the experiments and analyzed the data. K.Z., Z.G., R.D., and Z.L. performed the animal experiments and analyzed the data. M.Z., X.J., T.L., Y.C., and X.S. performed the molecular experiments. C.Z., W. Zhang, X.D., L.L., and W. Zhou conceived and directed the study and critically revised the manuscript. All authors approved the final version to be published.

DECLARATION OF INTERESTS

X.D. is a co-inventor of a patent entitled "AAV Vector Carrying ATP7B Gene Expression Frame and Variants and Its Application" (patent publication no. CN111088285B) and a co-founder of GeneCradle Therapeutics. Y.X. is an employee of GeneCradle Therapeutics. The drug structures used in this article have been authorized by the inventors and do not involve patent infringement issues.

SUPPLEMENTAL INFORMATION

Supplemental information can be found online at https://doi.org/10.1016/j.omtm.2025.101435.

REFERENCES

1. Bull, P.C., Thomas, G.R., Rommens, J.M., Forbes, J.R., and Cox, D.W. (1993). The Wilson disease gene is a putative copper transporting P-type ATPase similar to the Menkes gene. Nat. Genet. *5*, 327–337.
2. Petrukhin, K., Fischer, S.G., Pirastu, M., Tanzi, R.E., Chernov, I., Devoto, M., Brzustowicz, L.M., Cayanis, E., Vitale, E., Russo, J.J., et al. (1993). Mapping, cloning and genetic characterization of the region containing the Wilson disease gene. Nat. Genet. *5*, 338–343.
3. Tanzi, R.E., Petrukhin, K., Chernov, I., Pellequer, J.L., Wasco, W., Ross, B., Romano, D.M., Parano, E., Pavone, L., Brzustowicz, L.M., et al. (1993). The Wilson disease gene is a copper transporting ATPase with homology to the Menkes disease gene. Nat. Genet. *5*, 344–350.
4. Martínez-Morillo, E., and Bauça, J.M. (2022). Biochemical diagnosis of Wilson's disease: an update. Adv. Lab. Med. *3*, 103–125.
5. Alqahtani, S.A., Chami, R., Abuquteish, D., Vandriel, S.M., Yap, C., Kukkadi, L., Parmar, A., Mundh, A., Roberts, E.A., Kamath, B.M., and Siddiqui, I. (2022). Hepatic ultrastructural features distinguish paediatric Wilson disease from NAFLD and autoimmune hepatitis. Liver Int. *42*, 2482–2491.
6. Ala, A., Walker, A.P., Ashkan, K., Dooley, J.S., and Schilsky, M.L. (2007). Wilson's disease. Lancet *369*, 397–408.
7. Arai, S., Kogiso, T., Ogasawara, Y., Sagawa, T., Taniai, M., and Tokushige, K. (2021). Long-term outcome of Wilson's disease complicated by liver disease. JGH Open *5*, 793–800.
8. Alkhouri, N., Gonzalez-Peralta, R.P., and Medici, V. (2023). Wilson disease: a summary of the updated AASLD Practice Guidance. Hepatol. Commun. *7*, e0150.
9. Członkowska, A., Litwin, T., Dusek, P., Ferenci, P., Lutsenko, S., Medici, V., Rybakowski, J.K., Weiss, K.H., and Schilsky, M.L. (2018). Wilson disease. Nat. Rev. Dis. Primers *4*, 21.
10. Choi, W., Cha, S., and Kim, K. (2024). Navigating the CRISPR/Cas Landscape for Enhanced Diagnosis and Treatment of Wilson's Disease. Cell *13*, 1214.

11. Liu, S., Feng, Y., Huang, Y., Jiang, X., Tang, C., Tang, F., Zeng, C., and Liu, L. (2021). A GM1 gangliosidosis mutant mouse model exhibits activated microglia and disturbed autophagy. Exp. Biol. Med. *246*, 1330–1341.

12. Jiang, X., Liu, H., Shao, Y., Peng, M., Zhang, W., Li, D., Li, X., Cai, Y., Tan, T., Lu, X., et al. (2019). A novel GTPCH deficiency mouse model exhibiting tetrahydrobiopterin-related metabolic disturbance and infancy-onset motor impairments. Metabolism *94*, 96–104.

13. Cai, H., Cheng, X., and Wang, X.P. (2022). ATP7B gene therapy of autologous reprogrammed hepatocytes alleviates copper accumulation in a mouse model of Wilson's disease. Hepatology *76*, 1046–1057.

14. Hamilton, J.P., Koganti, L., Muchenditsi, A., Pendyala, V.S., Huso, D., Hankin, J., Murphy, R.C., Huster, D., Merle, U., Mangels, C., et al. (2016). Activation of liver X receptor/retinoid X receptor pathway ameliorates liver disease in Atp7B(-/-) (Wilson disease) mice. Hepatology *63*, 1828–1841.

15. Reed, E., Lutsenko, S., and Bandmann, O. (2018). Animal models of Wilson disease. J. Neurochem. *146*, 356–373.

16. Yoshida, M.C., Masuda, R., Sasaki, M., Takeichi, N., Kobayashi, H., Dempo, K., and Mori, M. (1987). New mutation causing hereditary hepatitis in the laboratory rat. J. Hered. *78*, 361–365.

17. Howell, J.M., and Mercer, J.F. (1994). The pathology and trace element status of the toxic milk mutant mouse. J. Comp. Pathol. *110*, 37–47.

18. Huster, D., Finegold, M.J., Morgan, C.T., Burkhead, J.L., Nixon, R., Vanderwerf, S.M., Gilliam, C.T., and Lutsenko, S. (2006). Consequences of copper accumulation in the livers of the Atp7b-/- (Wilson disease gene) knockout mice. Am. J. Pathol. *168*, 423–434.

19. Stremmel, W., and Weiskirchen, R. (2021). Therapeutic strategies in Wilson disease: pathophysiology and mode of action. Ann. Transl. Med. *9*, 732.

20. Roberts, E.A., and Schilsky, M.L. (2023). Current and Emerging Issues in Wilson's Disease. N. Engl. J. Med. *389*, 922–938.

21. Dev, S., Kruse, R.L., Hamilton, J.P., and Lutsenko, S. (2022). Wilson Disease: Update on Pathophysiology and Treatment. Front. Cell Dev. Biol. *10*, 871877.

22. Shribman, S., Webb, G., Taylor, R., Warner, T.T., Duckworth, A., Gimson, A., Shenoy, A., and Griffiths, W. (2020). Liver transplantation for late-onset presentations of acute liver failure in Wilson's disease: The UK experience over 2 decades. JHEP Rep. *2*, 100096.

23. Camarata, M.A., Ala, A., and Schilsky, M.L. (2019). Zinc Maintenance Therapy for Wilson Disease: A Comparison Between Zinc Acetate and Alternative Zinc Preparations. Hepatol. Commun. *3*, 1151–1158.

24. Litwin, T., Bembenek, J., Antos, A., Przybyłkowski, A., Skowrońska, M., Kurkowska-Jastrzębska, I., and Członkowska, A. (2022). Liver transplantation as a treatment for Wilson's disease with neurological presentation: a systematic literature review. Acta. Neurol. Belg. *122*, 505–518.

25. Poujois, A., Sobesky, R., Meissner, W.G., Brunet, A.S., Broussolle, E., Laurencin, C., Lion-François, L., Guillaud, O., Lachaux, A., Maillot, F., et al. (2020). Liver transplantation as a rescue therapy for severe neurologic forms of Wilson disease. Neurology *94*, e2189–e2202.

26. Stankiewicz, R., Patkowski, W., and Zieniewicz, K. (2021). Diagnostic Dilemma and Treatment Outcome in Acute Liver Failure Due to Wilson's Disease. Ann. Transplant. *26*, e930146.

27. Moini, M., To, U., and Schilsky, M.L. (2021). Recent advances in Wilson disease. Transl. Gastroenterol. Hepatol. *6*, 21.

28. Murillo, O., Luqui, D.M., Gazquez, C., Martinez-Espartosa, D., Navarro-Blasco, I., Monreal, J.I., Guembe, L., Moreno-Cermeño, A., Corrales, F.J., Prieto, J., et al. (2016). Long-term metabolic correction of Wilson's disease in a murine model by gene therapy. J. Hepatol. *64*, 419–426.

29. Murillo, O., Moreno, D., Gazquez, C., Barberia, M., Cenzano, I., Navarro, I., Uriarte, I., Sebastian, V., Arruebo, M., Ferrer, V., et al. (2019). Liver Expression of a MiniATP7B Gene Results in Long-Term Restoration of Copper Homeostasis in a Wilson Disease Model in Mice. Hepatology *70*, 108–126.

30. Leng, Y., Li, P., Zhou, L., Xiao, L., Liu, Y., Zheng, Z., Qin, F., Hao, Q., Xu, H., Yao, S., and Dong, B. (2019). Long-Term Correction of Copper Metabolism in Wilson's Disease Mice with AAV8 Vector Delivering Truncated ATP7B. Hum. Gene Ther. *30*, 1494–1504.

31. Greig, J.A., Nordin, J.M.L., Smith, M.K., Ashley, S.N., Draper, C., Zhu, Y., Bell, P., Buza, E.L., and Wilson, J.M. (2019). A Gene Therapy Approach to Improve Copper Metabolism and Prevent Liver Damage in a Mouse Model of Wilson Disease. Hum. Gene Ther. Clin. Dev. *30*, 29–39.

32. Padula, A., Spinelli, M., Nusco, E., Bujanda Cundin, X., Capolongo, F., Campione, S., Perna, C., Bastille, A., Ericson, M., Wang, C.C., et al. (2023). Genome editing without nucleases confers proliferative advantage to edited hepatocytes and corrects Wilson disease. JCI Insight *8*, e171281.

33. Mücke, M.M., Fong, S., Foster, G.R., Lillicrap, D., Miesbach, W., and Zeuzem, S. (2024). Adeno-associated viruses for gene therapy-clinical implications and liver-related complications, a guide for hepatologists. J. Hepatol. *80*, 352–361.

34. Sandahl, T.D., and Medici, V. (2022). Edging closer to successful gene therapy for Wilson disease. Mol. Ther. Methods Clin. Dev. *27*, 293–294.

35. Nathwani, A.C., McIntosh, J., and Sheridan, R. (2022). Liver Gene Therapy. Hum. Gene Ther. *33*, 879–888.

36. Guo, Y., Nyasae, L., Braiterman, L.T., and Hubbard, A.L. (2005). NH2-terminal signals in ATP7B Cu-ATPase mediate its Cu-dependent anterograde traffic in polarized hepatic cells. Am. J. Physiol. Gastrointest. Liver Physiol. *289*, G904–G916.

37. Maestro, S., Weber, N.D., Zabaleta, N., Aldabe, R., and Gonzalez-Aseguinolaza, G. (2021). Novel vectors and approaches for gene therapy in liver diseases. JHEP Rep. *3*, 100300.

38. Padula, A., Petruzzelli, R., Philbert, S.A., Church, S.J., Esposito, F., Campione, S., Monti, M., Capolongo, F., Perna, C., Nusco, E., et al. (2022). Full-length ATP7B reconstituted through protein *trans*-splicing corrects Wilson disease in mice. Mol. Ther. Methods Clin. Dev. *26*, 495–504.

39. Arjomandnejad, M., Dasgupta, I., Flotte, T.R., and Keeler, A.M. (2023). Immunogenicity of Recombinant Adeno-Associated Virus (AAV) Vectors for Gene Transfer. BioDrugs *37*, 311–329.

40. Duan, D. (2023). Lethal immunotoxicity in high-dose systemic AAV therapy. Mol. Ther. *31*, 3123–3126.

41. Cullis, P.R., and Hope, M.J. (2017). Lipid Nanoparticle Systems for Enabling Gene Therapies. Mol. Ther. *25*, 1467–1475.

42. Han, J.P., Lee, Y., Lee, J.H., Chung, H.Y., Lee, G.S., Nam, Y.R., Choi, M., Moon, K.S., Lee, H., Lee, H., and Yeom, S.C. (2023). In vivo genome editing using 244-cis LNPs and low-dose AAV achieves therapeutic threshold in hemophilia A mice. Mol. Ther. Nucleic Acids *34*, 102050.

43. Cheng, N., Wang, H., Wu, W., Yang, R., Liu, L., Han, Y., Guo, L., Hu, J., Xu, L., Zhao, J., et al. (2017). Spectrum of ATP7B mutations and genotype-phenotype correlation in large-scale Chinese patients with Wilson Disease. Clin. Genet. *92*, 69–79.

44. Lu, X., Li, S., Zhang, W., Lin, Y., Lu, Z., Cai, Y., Su, X., Shao, Y., Liu, Z., Sheng, H., et al. (2022). Assessment of the diagnostic value of serum ceruloplasmin for Wilson's disease in children. BMC Gastroenterol. *22*, 124.

45. Li, X., Lu, Z., Lin, Y., Lu, X., Xu, Y., Cheng, J., Shao, Y., Su, X., Liu, Z., Sheng, H., et al. (2019). Clinical features and mutational analysis in 114 young children with Wilson disease from South China. Am. J. Med. Genet. *179*, 1451–1458.

46. Yang, H., Liu, C.N., Wolf, R.M., Ralle, M., Dev, S., Pierson, H., Askin, F., Steele, K.E., Magnuson, T.H., Schweitzer, M.A., et al. (2019). Obesity is associated with copper elevation in serum and tissues. Metallomics *11*, 1363–1371.

47. Johncilla, M., and Mitchell, K.A. (2011). Pathology of the liver in copper overload. Semin. Liver Dis. *31*, 239–244.

48. Karadag, N., Tolan, K., Samdanci, E., Selimoglu, A., Akpolat, N., and Yilmaz, S. (2017). Effect of Copper Staining in Wilson Disease: A Liver Explant Study. Exp. Clin. Transplant. *15*, 542–546.

OMTM, Volume *33*

Supplemental information

Evaluation of efficacy and safety of AAV8-ΔC4ATP7B gene therapy in a mutant mouse model of Wilson's disease

Chunhua Zeng, Yunting Lin, Xinshuo Lu, Shehong Chen, Yan Xia, Kangdi Zhang, Yongxian Shao, Zhihong Guan, Rong Du, Zongcai Liu, Mingqi Zhao, Xiaoling Jiang, Yanna Cai, Taolin Li, Xueying Su, Yaoyong Chen, Xiaoyan Dong, Wen Zhang, Li Liu, and Wenhao Zhou

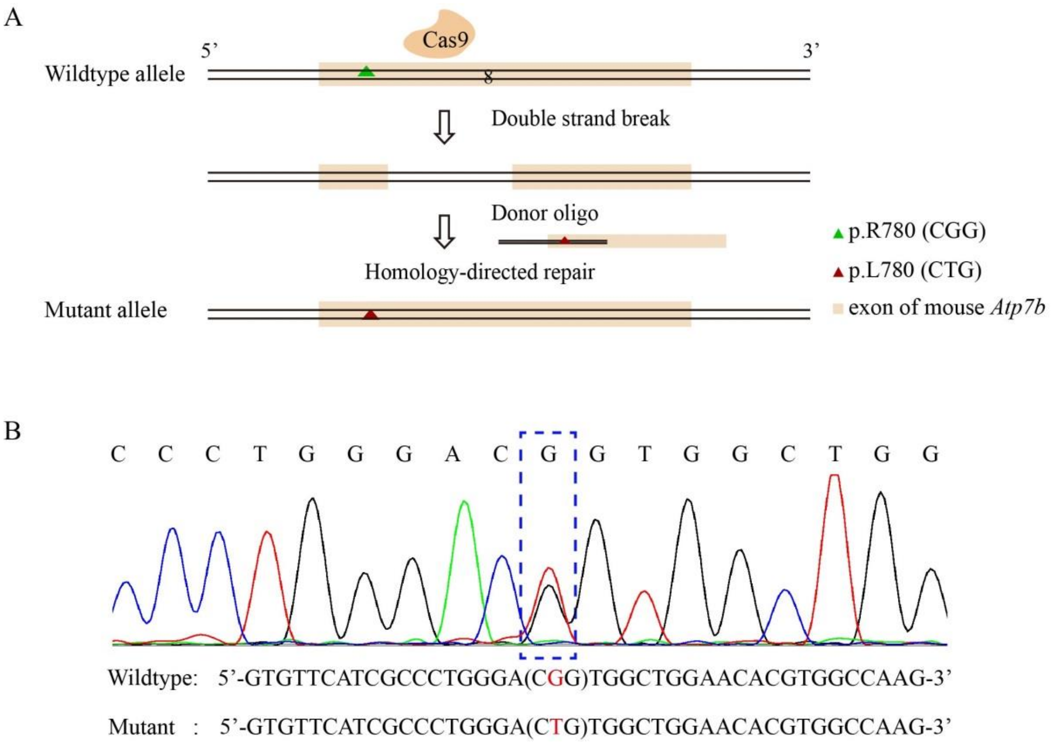

Figure S1. Generation of a C57BL/6 mouse model with *Atp7b*$^{\mathbf{R780L}}$ variant.

(A) Schematic diagram on the generation of the *Atp7b*$^{KI/KI}$ mouse model. (B) Confirmation of the *Atp7b*^{R780L} variant by Sanger sequencing.

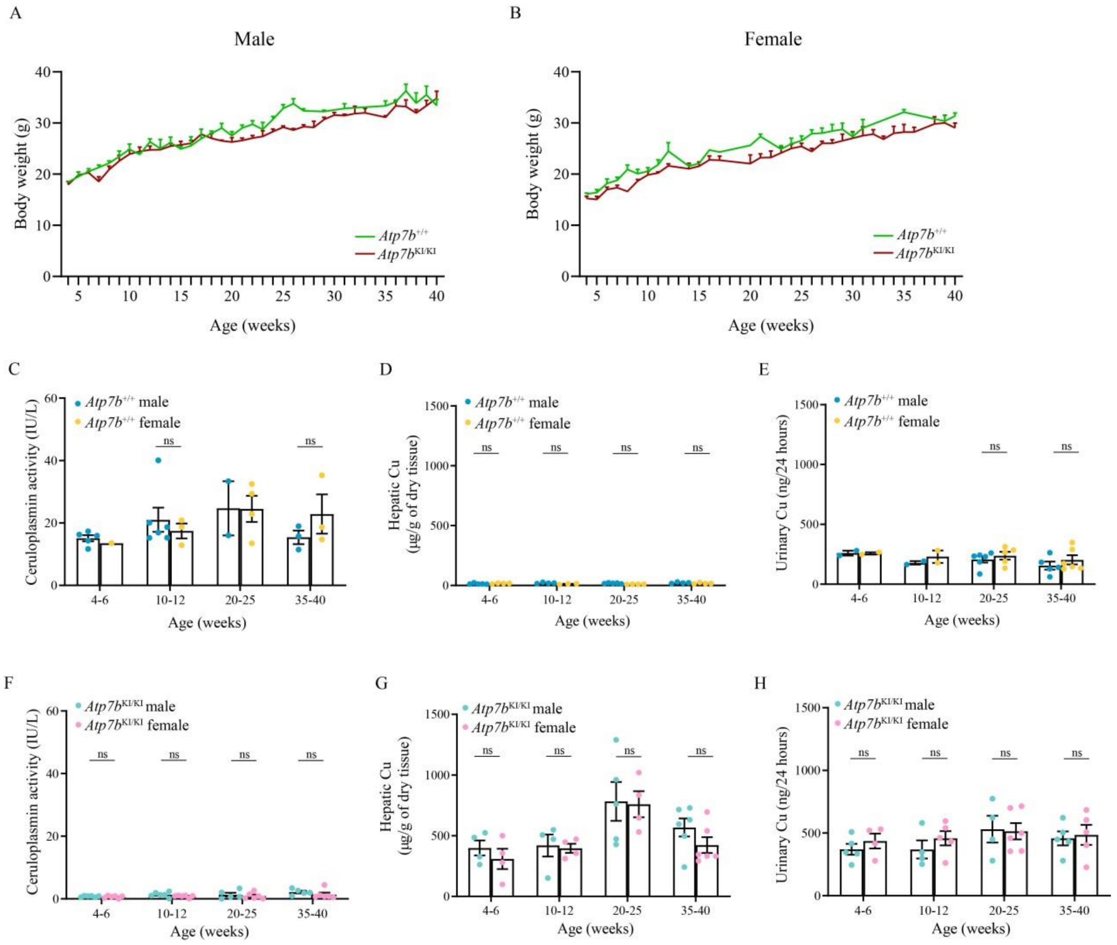

Figure S2. The weight gain and Cu metabolism biomarkers of *Atp7b*$^{KI/KI}$ and *Atp7b*$^{+/+}$ mice.

(A) Body weight in male *Atp7b*$^{KI/KI}$ and *Atp7b*$^{+/+}$ mice. (B) Body weight in female *Atp7b*$^{KI/KI}$ and *Atp7b*$^{+/+}$ mice. (C) Serum ceruloplasmin levels in *Atp7b*$^{+/+}$ mice. (D) Hepatic Cu content in *Atp7b*$^{+/+}$ mice. (E) Urinary Cu levels in *Atp7b*$^{+/+}$ mice. (F) Serum ceruloplasmin levels in *Atp7b*$^{KI/KI}$ mice. (G) Hepatic Cu content in *Atp7b*$^{KI/KI}$ mice. (H) Urinary Cu levels in *Atp7b*$^{KI/KI}$ mice.

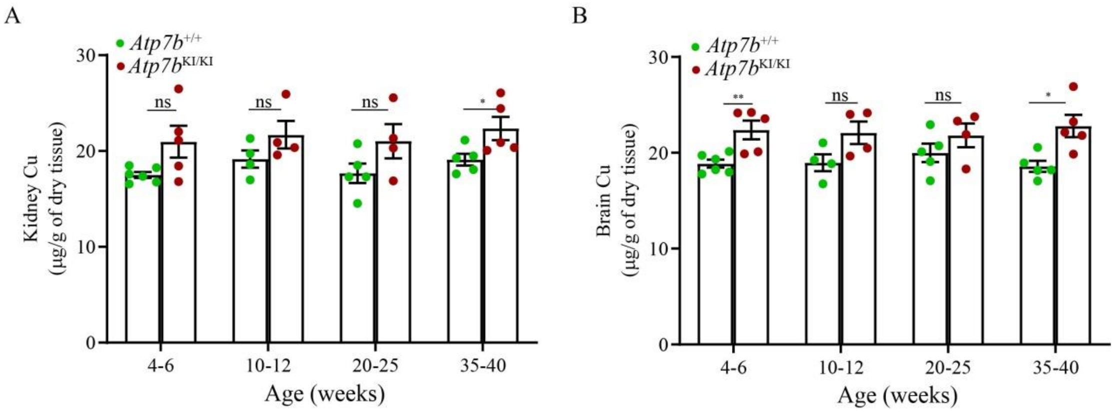

Figure S3. Kidney and brain Cu content in *Atp7b*$^{KI/KI}$ mice.

(A) Comparison of kidney Cu content between *Atp7b*$^{KI/KI}$ and *Atp7b*$^{+/+}$ mice. (B) Comparison of brain Cu content between *Atp7b*$^{KI/KI}$ and *Atp7b*$^{+/+}$ mice.

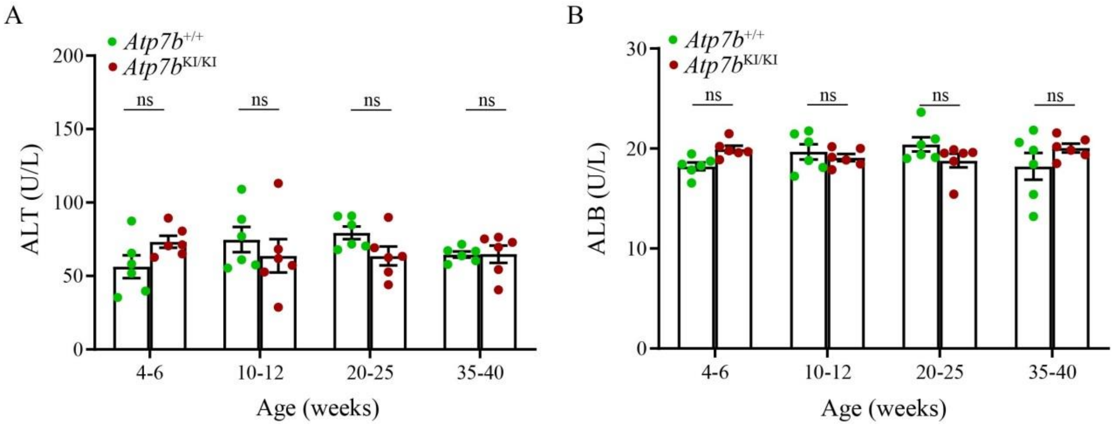

Figure S4. Liver functions of *Atp7b*$^{KI/KI}$ and *Atp7b*$^{+/+}$ mice

(A) Comparison of serum ALT levels between *Atp7b*$^{KI/KI}$ and *Atp7b*$^{+/+}$ mice. (B) Comparison of serum ALB levels between *Atp7b*$^{KI/KI}$ and *Atp7b*$^{+/+}$ mice.

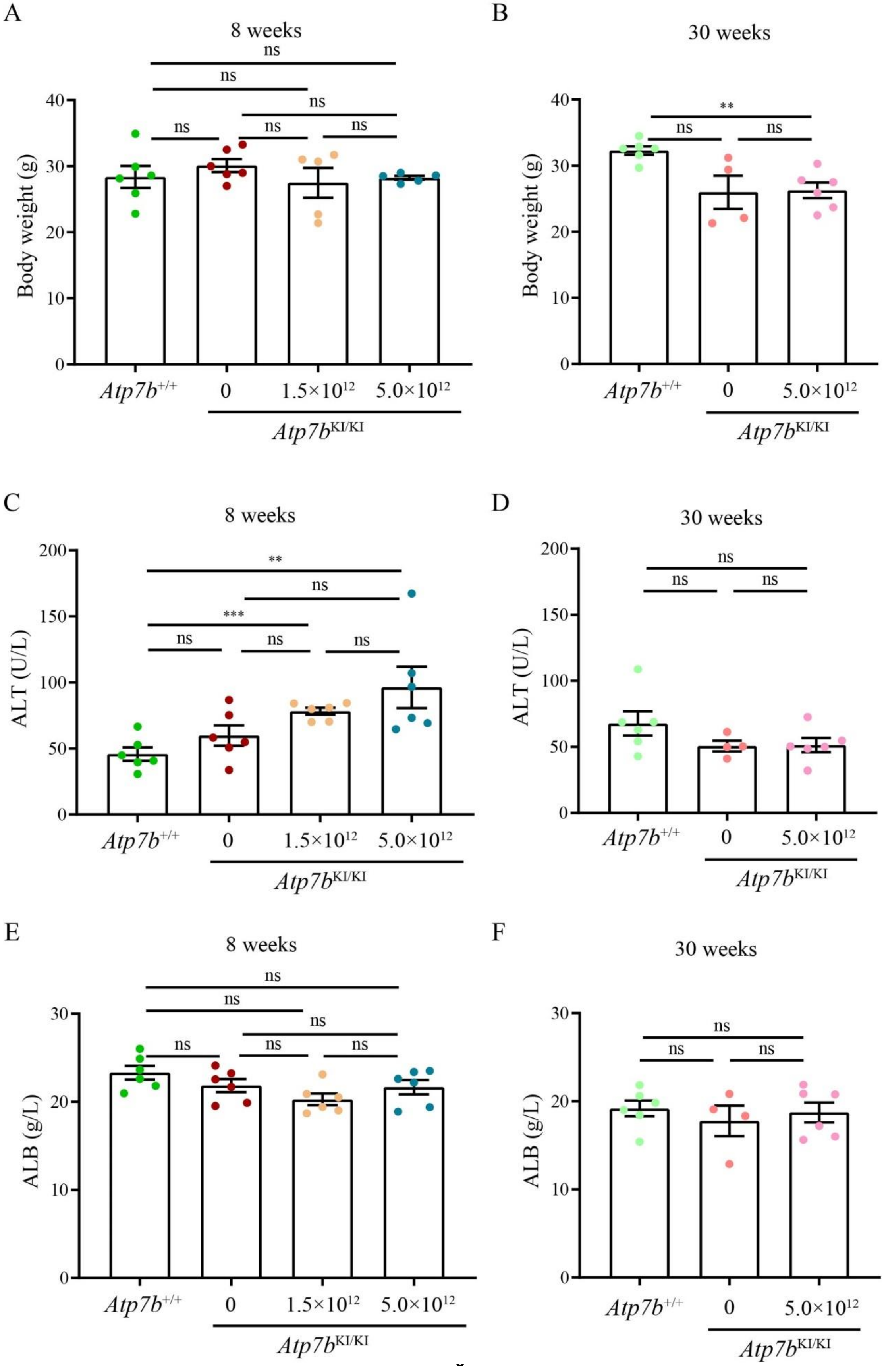
A
8 weeks
ns
ns
ns
ns
ns
ns
Body weight (g)
0
10
20
30
40
Atp7b+/+
0
1.5×10^12
5.0×10^12
Atp7bKI/KI
B
30 weeks
**
ns
ns
Body weight (g)
0
10
20
30
40
Atp7b+/+
0
5.0×10^12
Atp7bKI/KI
C
8 weeks
**
ns
***
ns
ns
ns
ALT (U/L)
0
50
100
150
200
Atp7b+/+
0
1.5×10^12
5.0×10^12
Atp7bKI/KI
D
30 weeks
ns
ns
ns
ALT (U/L)
0
50
100
150
200
Atp7b+/+
0
5.0×10^12
Atp7bKI/KI
E
8 weeks
ns
ns
ns
ns
ns
ns
ALB (g/L)
0
10
20
30
Atp7b+/+
0
1.5×10^12
5.0×10^12
Atp7bKI/KI
F
30 weeks
ns
ns
ns
ALB (g/L)
0
10
20
30
Atp7b+/+
0
5.0×10^12
Atp7bKI/KI

Figure S5. The body weight and liver functions in *Atp7b*$^{KI/KI}$ mice after AAV8-Δ C4ATP7B therapy.

(A) Comparison of body weight among treated *Atp7b*$^{KI/KI}$, untreated *Atp7b*$^{KI/KI}$, and *Atp7b*$^{+/+}$ mice at 8 weeks after treatment. (B) Comparison of body weight among treated *Atp7b*$^{KI/KI}$, untreated *Atp7b*$^{KI/KI}$, and *Atp7b*$^{+/+}$ mice at 30 weeks after treatment. (C) Comparison of serum ALT levels among treated *Atp7b*$^{KI/KI}$, untreated *Atp7b*$^{KI/KI}$, and *Atp7b*$^{+/+}$ mice at 8 weeks after treatment. (D) Comparison of serum ALT levels among treated *Atp7b*$^{KI/KI}$, untreated *Atp7b*$^{KI/KI}$, and *Atp7b*$^{+/+}$ mice at 30 weeks after treatment. (E) Comparison of serum ALB levels among treated *Atp7b*$^{KI/KI}$, untreated *Atp7b*$^{KI/KI}$, and *Atp7b*$^{+/+}$ mice at 8 weeks after treatment. (F) Comparison of serum ALB levels among treated *Atp7b*$^{KI/KI}$, untreated *Atp7b*$^{KI/KI}$, and *Atp7b*$^{+/+}$ mice at 30 weeks after treatment.

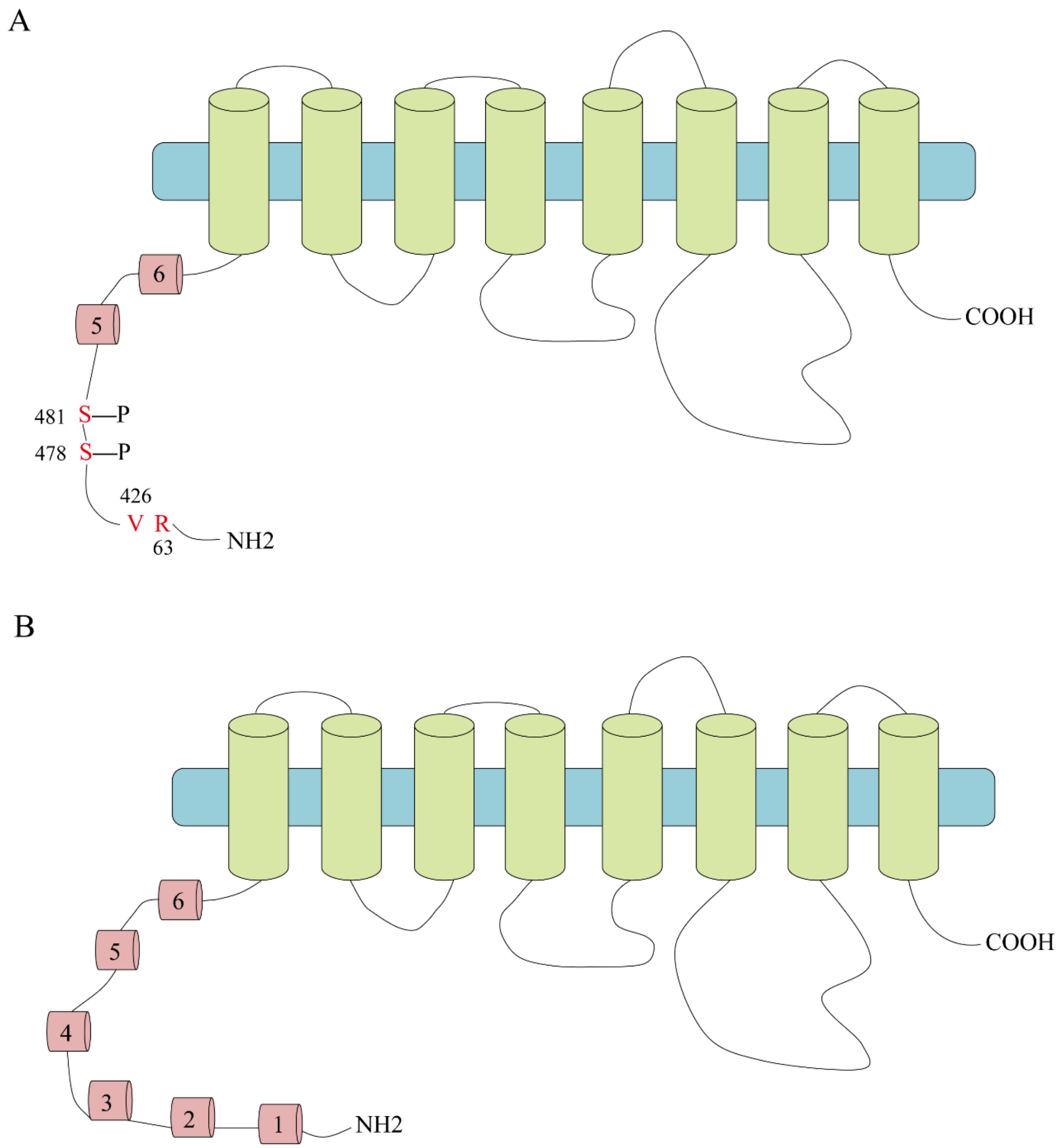

Figure S6. Reconstruction of truncated human ATP7B (ΔC4ATP7B) proteins.

(A) Schematic representation of truncated ATP7B (ΔC4ATP7B) proteins. In ΔC4ATP7B protein, the first 4 amino-terminal metal-binding domains (pink boxes) have been eliminated, 63 amino acids at the N-terminal have been preserved and the sequence containing the ser478 and ser481 phosphorylation sites between MBD4-5 have been retained. (B) Schematic representation of full-length ATP7B proteins.

Table S1. Study design for evaluation of AAV8-ΔC4ATP7B gene therapy.

Group	Genotype	N	Gender	Age (weeks)	Dose of AAV8-ΔC4ATP7B (vg/kg)	Sacrifice time after treatment (weeks)
1 (control)	*Atp7b*$^{+/+}$	6	3M/3F	10	0	8
2 (control)	*Atp7b*$^{KI/KI}$	6	3M/3F	10	0	8
3 (treatment)	*Atp7b*$^{KI/KI}$	6	5M/1F	10	1.5×10^{12}	8
4 (treatment)	*Atp7b*$^{KI/KI}$	6	5M/1F	10	5.0×10^{12}	8
5 (control)	*Atp7b*$^{+/+}$	6	3M/3F	10	0	30
6 (control)	*Atp7b*$^{KI/KI}$	6	3M/3F	10	0	30
7 (treatment)	*Atp7b*$^{KI/KI}$	6	3M/3F	10	5.0×10^{12}	30

N, number; M, male; F, female

Table S2. Primer sequences for qPCR assays.

Gene	Forward primer (5'→3')	Reverse primer (5'→3')
GAPDH	AGGTCGGTGTGAACGGATTTG	GGGGTCGTTGATGGCAACA
ΔC4ATP7B	AGAGCTTCGCCTTTGACAAC	TCTCAGACACCACTCTCACG
AAV vector	TACCTTTTTTACAGGGTACCGCCACCATGC	TCAGACACCACTCTCACGGTGGAGGT
